# A DFT study on the C–H oxidation reactivity of Fe(iv)–oxo species with N4/N5 ligands derived from l-proline†

**DOI:** 10.1039/d0ra08496d

**Published:** 2021-01-08

**Authors:** Jin Lin, Qiangsheng Sun, Wei Sun

**Affiliations:** State Key Laboratory for Oxo Synthesis and Selective Oxidation, Center for Excellence in Molecular Synthesis, Suzhou Research Institute of LICP, Lanzhou Institute of Chemical Physics (LICP), Chinese Academy of Sciences Lanzhou 730000 P. R. China wsun@licp.cas.cn; University of Chinese Academy of Sciences Beijing 100049 P. R. China

## Abstract

The hydroxylation of hexane by two Fe^IV^O complexes bearing a pentadentate ligand (N5, Pro3Py) and a tetradentate ligand (N4, Pro2PyBn) derived from l-proline was studied by DFT calculations. Theoretical results predict that both Fe^IV^O complexes hold triplet ground states. The hydrogen atom abstraction (HAA) processes by both Fe^IV^O species proceed through a two-state reactivity, thus indicating that HAA occurs *via* a low-barrier quintet surface. Beyond the conventional rebound step, the dissociation path is also calculated and is found to potentially occur after HAA.

## Abstract 2

The hydroxylation of hexane by two Fe^IV^O complexes bearing a pentadentate ligand (N5, Pro3Py) and a tetradentate ligand (N4, Pro2PyBn) derived from l-proline was studied by DFT calculations.

## Graphical abstract

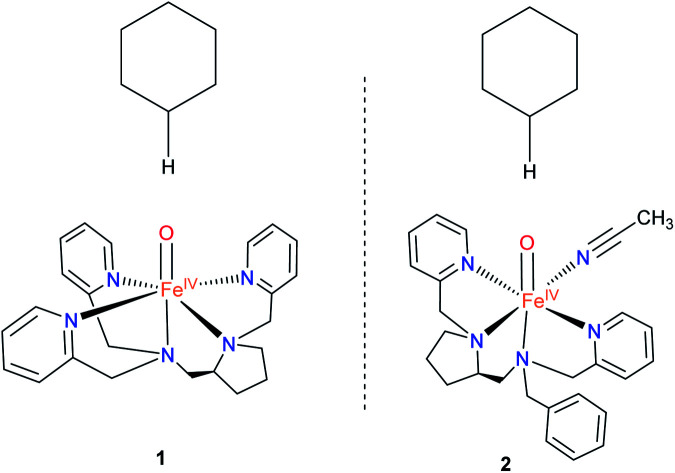

## Introduction

**Scheme 1:**
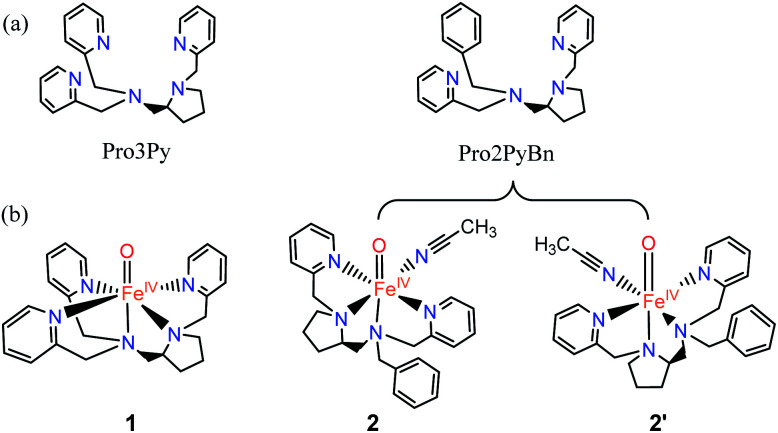
(a) Pentadentate ligand (N5, Pro3Py) and the tetradentate ligand (N4, Pro2PyBn). (b) Fe^IV^O species: [Pro3PyFe^IV^(O)]^2+^1, [Pro2PyBnFe^IV^(O) (CH_3_CN)]^2+^2 and its isomer 2′.

Non-haem iron enzymes and their synthetic analogues have attracted considerable interest due to their ability for diverse catalytic oxidation in biological and chemical syntheses.^1–5^ In these oxidative transformations, Fe(iv)=O species are often implicated as key oxidizing intermediates.^6–10^ Of crucial interest is the effect of the spin state of the Fe^IV^(O) unit; quintet (*S* = 2) or triplet (*S* = 1) ground states might be involved as predicted by density functional theory (DFT) calculations.^11,12^ For instance, a high-spin iron(iv)–oxo intermediate was spectroscopically characterized in α-ketoglutarate-dependent taurine dioxygenase (TauD).^13^ Additionally, the first X-ray crystal structure of a synthetic non-haem iron(iv)–oxo complex, namely [(TMC)Fe^IV^(O)]^2+^ (TMC = 1,4,8,11-tetramethyl-1,4,8,11-tetraazacyclotetradecane), was reported in 2003.^14^ Inspired by these great achievements in both biological and biomimetic systems, a great number of mononuclear non-haem Fe(iv)=O complexes supported by polydentate ligands have been synthesized and intensively investigated to elucidate their structural, spectroscopic and reactive properties.^6–10,15–19^ These Fe(iv)=O complexes exhibit versatile reactivity (for example, enabling alkane hydroxylation) that markedly depends on the use of supporting ligands.^16^ In recent years, the Fe(iv)=O complex with a Me_3_NTB (tris(*N*-methylbenzimidazol-2-yl)-methyl)amine) ligand has been demonstrated as the most powerful oxidant among the intermediate-spin (*S* = 1) iron(iv)–oxo complexes.^20^ Moreover, a highly reactive high-spin (*S* = 2) complex [(TQA)Fe^IV^(O) (CH_3_CN)]^2+^ (TQA = tris(quinolin-2-ylmethyl)amine) was prepared by replacing the pyridines of the TPA ligand with weaker-field quinolones.^21^ This intriguing difference in the spin state is strongly regulated by the structure of the supporting ligand, thus tuning the reactivity in the activation of C–H bonds.^22^ On the other hand, DFT studies have indicated a small energy gap in oxo–iron(iv) models between triplet and quintet ground states, thus enabling “two-state-reactivity” (TSR).^23,24^ For example, during the reaction of [N_4_PyFe^IV^(O)]^2+^ with cyclohexane, the triplet ground state displays a high activation barrier in the hydrogen atom abstraction (HAA) step, while the reaction proceeds *via* the quintet surface with a much lower barrier.^23–25^ Nevertheless, a broader analysis is needed in computational studies. Recently, we have prepared a series of linear polydentate nitrogen ligands, and their iron and manganese complexes have demonstrated an excellent performance in the enantioselective oxidation of C=C and C–H bonds.^26^ Among these ligands, two derived from l-proline, namely Pro3Py (pentadentate ligand, N5) and Pro2PyBn (tetradentate ligand, N4), have similar structures (Scheme 1).^27–29^ Herein, we report calculations on the hydroxylation of cyclohexane (CYH) by synthetic non-haem Fe^IV^O complexes with both N4/N5 supporting ligands (Pro3Py and Pro2PyBn ligands, Scheme 1). To understand the selectivity between rebound and dissociation, the substrate radical dissociation step after HAA by both Fe^IV^O species is also calculated with DFT.

## Calculation method

All DFT calculations were performed with the Gaussian 16 suite of quantum chemical packages.^30^ The spin-unrestricted B3LYP functional^31–34^ corrected with Grimme's D3 dispersion and Becke–Johnson damping,^35,36^ UB3LYP-D3(BJ), was used in all calculations. Two basis sets were employed: (i) SDD^37^ for the Fe atom and 6-31G*^38^ for remaining atoms. This basis set is denoted as B1 and is used to optimize transition states and minima. (ii) The Def2-TZVPP^39^ basis set for all atoms, denoted as B2, is used for single-point energy corrections. Transition states were ascertained by vibrational frequency analysis to possess only one imaginary frequency. All optimizations and single-point calculations including solvation were performed using the self-consistent reaction field (SCRF) calculations in the polarizable continuum model (PCM); the experimental solvent acetonitrile (*ε* = 35.688)^40,41^ was used. All geometries were fully optimized without symmetry. Frequency calculations were performed to ascertain that minima had no imaginary frequency and transition states had only one imaginary frequency. The spin density iso-surfaces were plotted using the Multiwfn software.^42^ Energies in the following text were the electronic energies corrected by zero-point vibrational energy (−20 °C) at the UB3LYP-D3(BJ)/B1 level.

## Results and discussion

### Geometry of 1 and 2

**Fig. 1:**
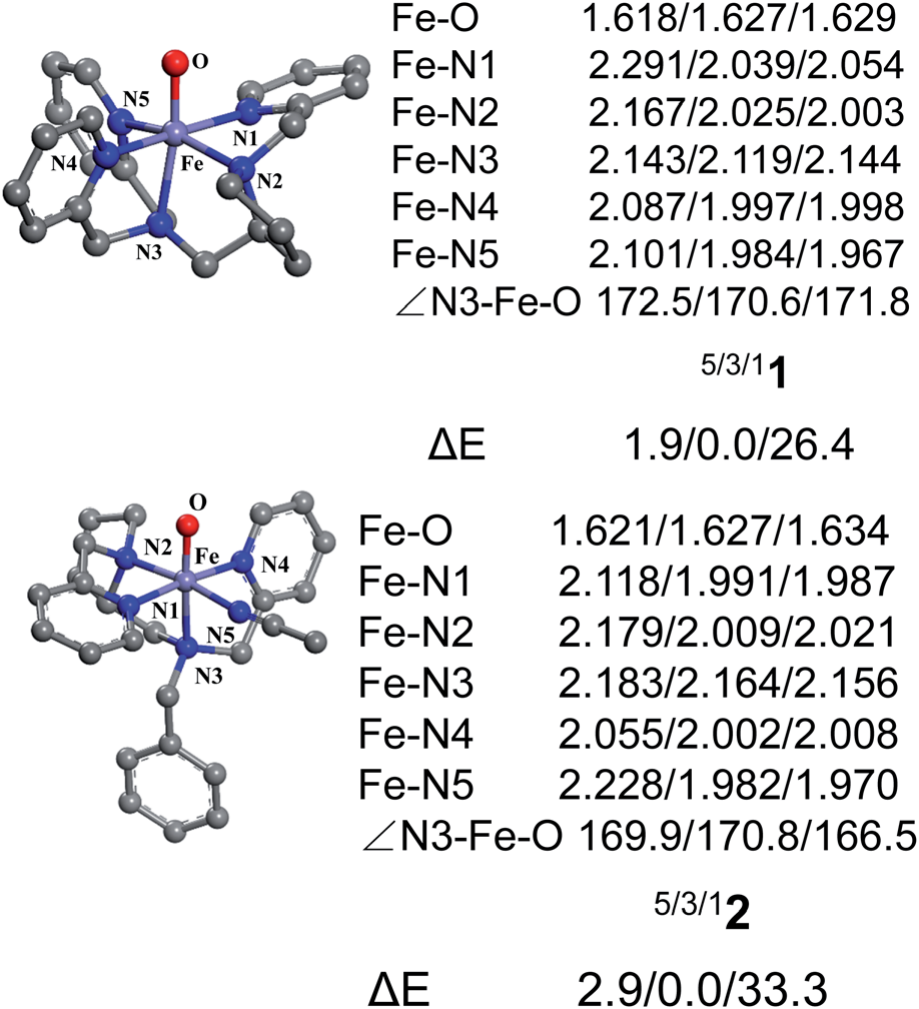
Key geometric parameters for [Pro3PyFe^IV^(O)]^2+^1 and [Pro2PyBnFe^IV^(O) (CH_3_CN)]^2+^2 at the UB3LYP-D3(BJ)/B1 level and spin-state energy gaps at the UB3LYP-D3(BJ)/B2//B1 level (including ZPE and solvation corrections).

Fig. 1 shows the key geometric parameters and spin-state energy gaps of [Pro3PyFe^IV^(O)]^2+^1 and [Pro2PyBnFe^IV^(O) (CH_3_CN)]^2+^2. In particular, 1 contains two pyridine rings parallel to the Fe–O axis, and the third ring is perpendicular to the Fe–O axis. For the N4 ligand Pro2PyBn, Fe^IV^(O) species 2 contains one pyridine ring parallel to Fe–O, and the incoming solvent acetonitrile acts as a sixth ligand. In addition, Fe^IV^(O) species 2 has an isomer 2′, in which the oxo moiety is trans to the nitrogen of pyrrolidine (Scheme 1b). On the basis of DFT calculations at the UB3LYP-D3(BJ)/B1 level, these Fe^IV^(O) complexes (1, 2 and 2′) have triplet ground states and low-lying quintet excited states, consistent with previous reports. The bond length of Fe–O is 1.627 Å in both ^3^1 and ^3^2, which is approximately 0.2 Å shorter than that of triplet [Fe^IV^(O) (TMC) (CH_3_CN)]^2+^ (1.646 Å)^3^ and 0.1 Å shorter than that of triplet [Fe^IV^(O) (N4Py)]^2+^ (1.639 Å).^23–25,43^ The energies of singlet spin states 1 and 2 are very high and thus can be ruled out in the C–H activation reaction.

### H-Atom abstraction reactivity of 1 and 2 with cyclohexane

**Fig. 2:**
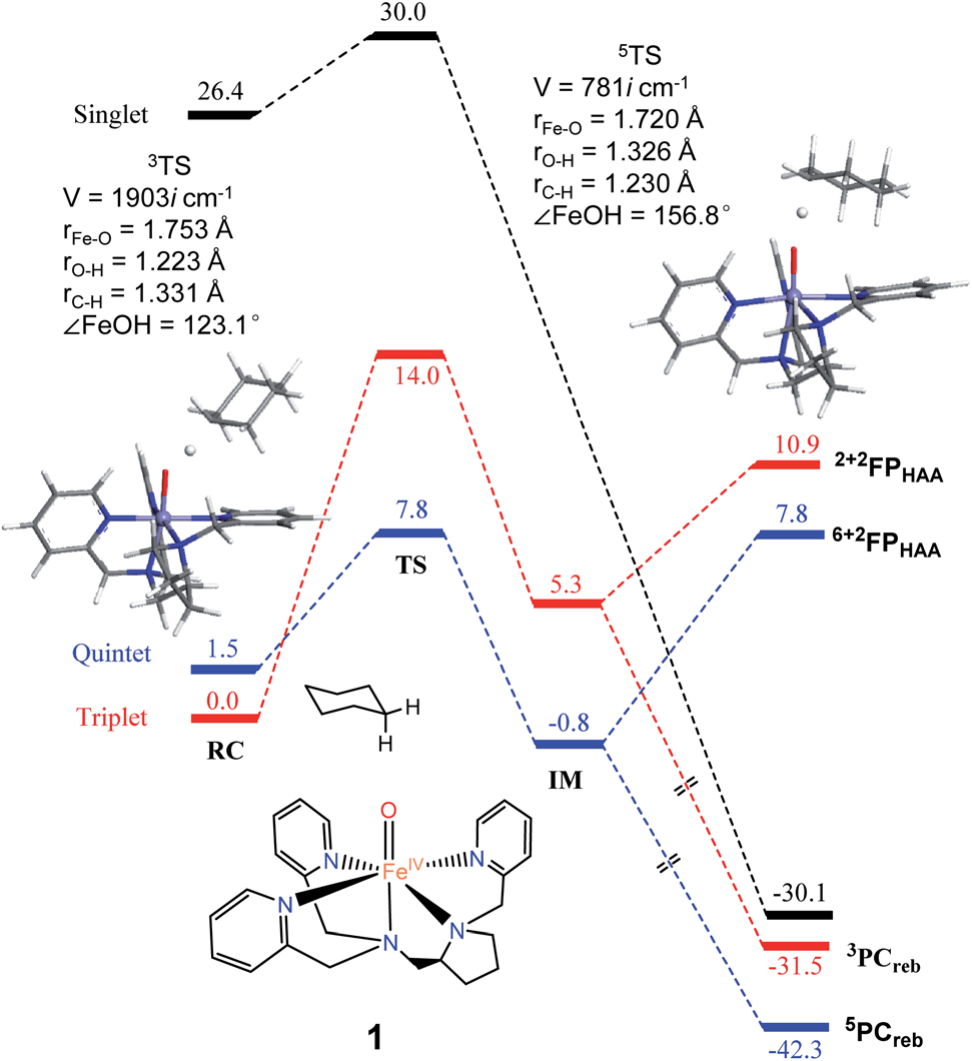
The key geometric parameters of transition state structures and energy profile for cyclohexane hydroxylation by 1 at the UB3LYP-D3(BJ)/B2//B1 level with solvent correction. RC: reactant cluster, TS: transition state, IM: intermediate, PC_reb_: rebound production, FP: free production after H-atom abstraction.

**Fig. 3:**
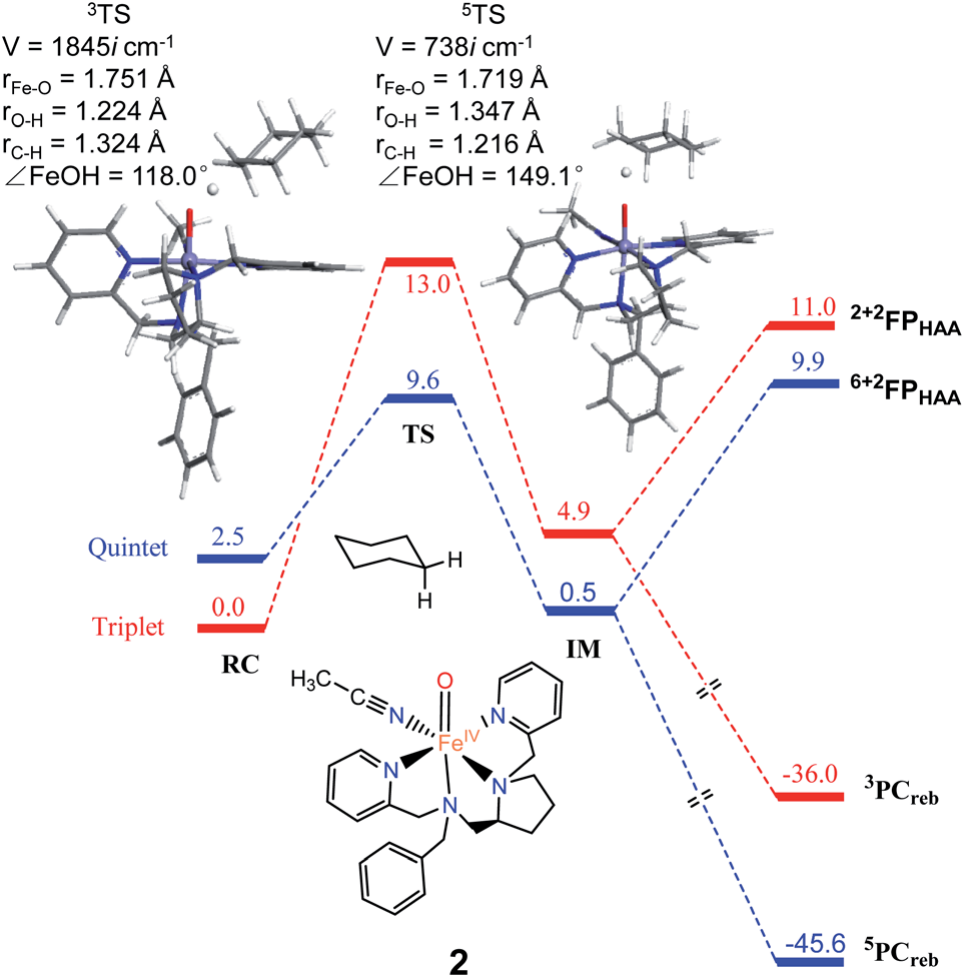
The key geometric parameters of transition state structures and energy profile for cyclohexane hydroxylation by 2 at the UB3LYP-D3(BJ)/B2//B1 level with solvent correction. RC: Reactant cluster, TS: transition state, IM: intermediate, PC_reb_: rebound productions, FP: free productions after H-atom abstraction.

As shown in Fig. 2, H-atom abstraction is the rate-determining step in cyclohexane hydroxylation by [Pro3PyFe^IV^(O)]^2+^1, which is in accordance with experimental findings.^29^ During the Fe–O bond elongation, spin reversion takes place, thus switching the reaction pathway from triplet to quintet spin states in the transition state. For [Pro3PyFe^IV^(O)]^2+^1, barriers in triplet and quintet states are found to be 14.0 and 7.8 kcal mol^−1^, respectively, thus making the quintet state more accessible in the HAA process. This clearly indicates that a TSR process is involved in the activation of the C–H bond by 1, following well-established patterns shown by non-haem synthetic Fe^IV^O species.^23–25^ In the case of 2 [Pro2PyBnFe^IV^(O) (CH_3_CN)]^2+^, the same trends are also observed based on the calculated data (Fig. 3), wherein barriers in the triplet and quintet states are 13.0 and 9.6 kcal mol^−1^, respectively. As for isomer 2′, barriers are almost the same as 2 (9.4 kcal mol^−1^, shown in Fig. S4†). In comparison, [Pro3PyFe^IV^(O)]^2+^1 seems to be more reactive than [Pro2PyBnFe^IV^(O) (CH_3_CN)]^2+^2 and 2′. Following the HAA process, IMs (Fig. 2 and 3) from both 1 and 2 rebound to form alcohol complexes without any distinct barriers in both triplet and quintet states.

**Scheme 2:**
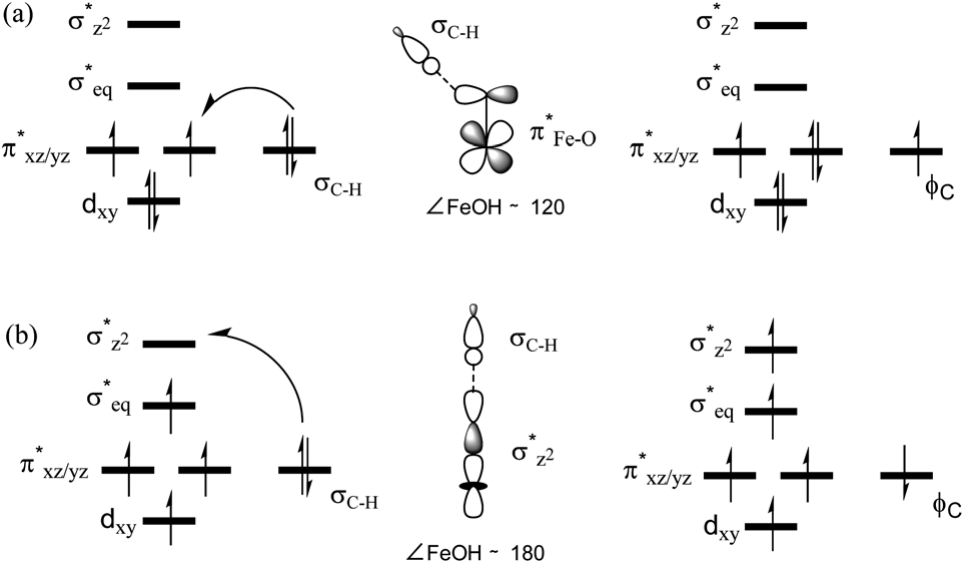
Electron evolution diagrams during the conversion of (a) ^3^RC to ^3^IM and (b) ^5^RC to ^5^IM and orbital overlap cartoons in the middle panel.

**Fig. 4:**
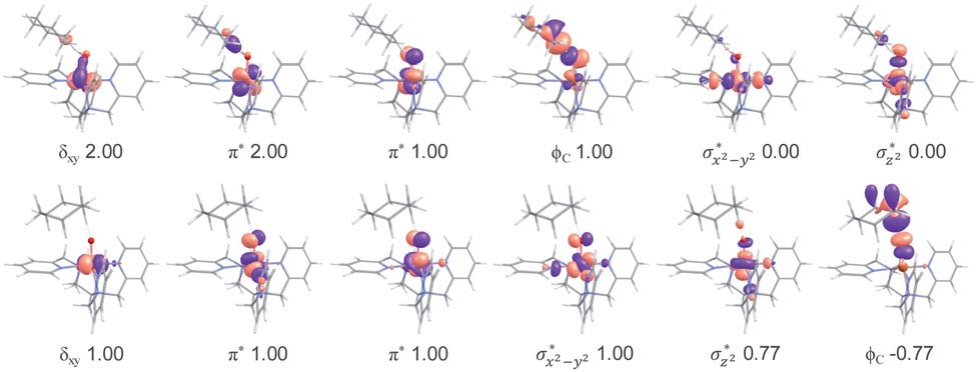
Natural d orbitals and their occupations in ^3^TS (top panel) and spin natural orbitals and their occupations in ^5^TS (bottom panel) for the H-abstraction reaction of 1 with cyclohexane.

**Fig. 5:**
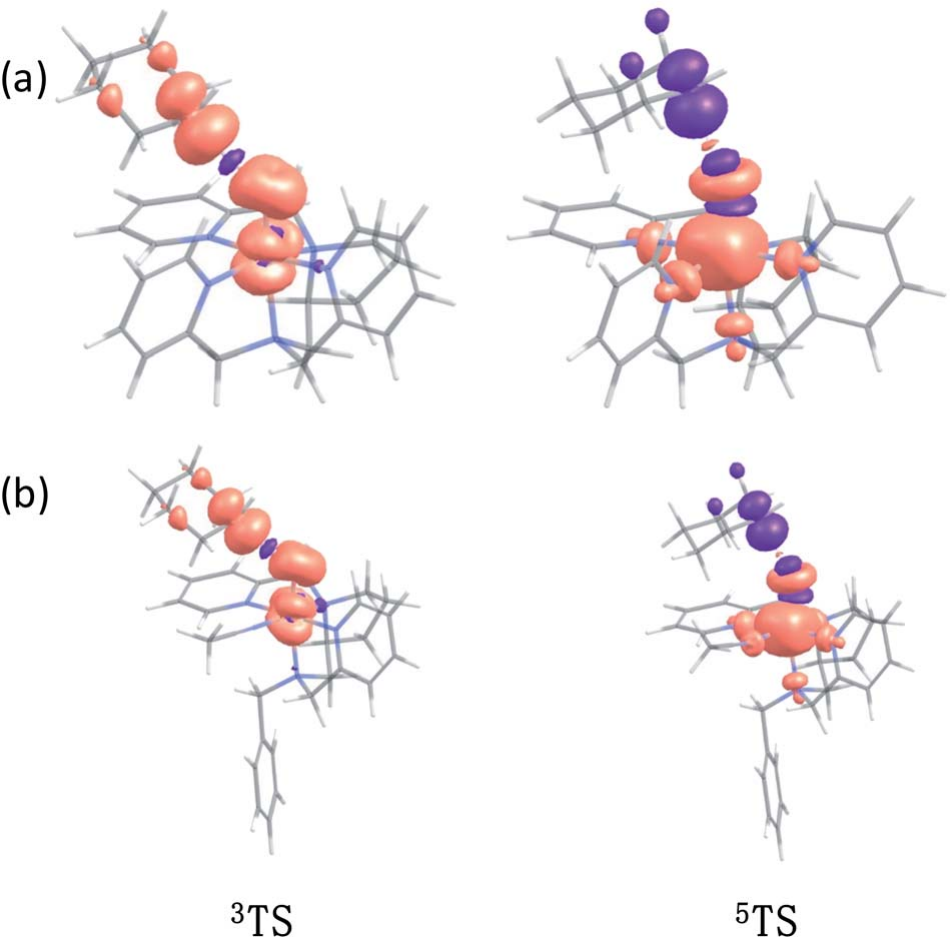
The spin density plot of (a) ^3^TS, ^5^TS for CYH + 1; (b) ^3^TS, ^5^TS for CYH + 2 contour value = 0.005.

In addition to the reaction pathway, electronic structures involved in the HAA step are also of great interest. Lower energies on the quintet surface for both Fe^IV^O species 1 and 2 follow well-established patterns shown by previously reported non-haem synthetic Fe^IV^O complexes.^23–25^ Electron shift diagrams are shown in Scheme 2 for triplet and quintet spin states. In Scheme 2a, the triplet sideway trajectory, a β-spin electron shifts to the π*_xz/*yz*_ d orbital, producing doublet Fe^III^OH coupled to the α-spin electron of the radical substrate (in *ϕ*_C_). In the quintet upright trajectory, α-spin electron from the substrate shifts to the σ*_z^2^_ orbital of the Fe^IV^O moiety, produces a β-spin radical substrate, thereby strengthening stabilizing exchange interactions with other unpaired electrons. This is a type of exchange-enhanced reactivity (EER).^11,44^ According to the model of orbital overlap at TS (Scheme 2 middle panel), these sideway or upright trajectories are also called π paths or σ paths. Note that TS has the same configuration as IM, and spin natural orbitals and natural orbitals for quintet and triplet TS are shown in Fig. 4 and S2,† respectively. For ^3^TS, the *ϕ*_C_ orbital contains a small amount of π* of the FeO moiety; the number of electrons in this *ϕ*_C_ is 1.00. However, the occupation of *ϕ*_C_ becomes negative in ^5^TS, and the *ϕ*_C_ orbital contains a small amount of σ*_z^2^_ of the FeO moiety. Moreover, the spin density plot (Fig. 5) also shows that CYH has the opposite spin density in ^3^TS and ^5^TS. These pictures directly reflect the difference of electron shift in triplet and quintet H-abstraction process.

The Mulliken charge of H becomes more positive during H-abstraction, but no distinct changes of Mulliken spin in H is observed (Table S2†), which indicates a proton transformation. Inversely, no changes are found in the Mulliken charge of the CYH radical substrate, and the spin of the CYH radical substrate becomes more positive at the triplet and more negative at the quintet. These data indicate that the H-abstraction and electron shift are concerted proton-coupled electron transfer (PCET) processes. It is consistent with previously reported results.^25,45–47^

### Post H-atom abstraction

In biological enzymes and biomimetic models, the C–H bond activation of alkanes by Fe^IV^O species has been well-documented to give alcohol products *via* the HAA/oxygen rebound mechanism.^48^ Beyond the rebound step, instead, the substrate radical may escape from the cage and go through the dissociation mechanism.^49,50^ The non-rebound pathway has been observed in some non-haem Fe^IV^O complexes, such as [N_4_PyFe^IV^(O)]^2+^ and [(Bn-TPEN)-Fe^IV^O]^2+^.^18,25^ Based on experimental and theoretical studies, the dissociation of the substrate radical has been shown to be feasible. In the present study, as shown in Fig. 2, 3 and S4,† energies for radical dissociation are not much higher than the activation energy of H-abstraction, which means that the dissociation mechanism might occur after the HAA step.^25^

## Conclusions

In conclusion, DFT calculations at the UB3LYP-D3(BJ)/Def2-TZVPP//SDD/6-31G* level have been carried out to study the hydroxylation of hexane by two Fe^IV^O complexes bearing a pentadentate ligand (N5, Pro3Py) and a tetradentate ligand (N4, Pro2PyBn) derived from l-proline. Theoretical results have revealed the activation of the C–H bond by Fe^IV^O species 1 (N5) and 2 (N4) *via* a TSR process, thus making HAA occur in a lower barrier at the quintet spin state. These computational predictions are in agreement with well-documented [N_4_PyFe^IV^(O)]^2+^ species.^23–25^ On the basis of theoretical data, the dissociation of substrate radicals formed after HAA by both Fe^IV^O species is possible. Additionally, the EER principle predicts the dominance of the quintet spin state during the entire reaction of the C–H hydroxylation of cyclohexane.

## Conflicts of interest

There are no conflicts to declare.

## Supplementary Material

RA-011-D0RA08496D-s001
