# Supplementary material for: A DFT study on the C–H oxidation reactivity of Fe(iv)–oxo species with N4/N5 ligands derived from l-proline
Source: RSC Adv. 2021 Jan 8;11(4):2293–7. doi: 10.1039/d0ra08496d (PMC8693871; doi:10.1039/d0ra08496d)
Supplement: RA-011-D0RA08496D-s001 [file RA-011-D0RA08496D-s001.pdf]

Electronic Supplementary Material (ESI) for RSC Advances.
This journal is © The Royal Society of Chemistry 2020

DFT study on C-H oxidation reactivity of Fe(IV)-oxo species with N4/N5 ligandsderived from L-proline

Jin Lin,[ab] Qiangsheng Sun,[a] Wei Sun*[a]

[a] State Key Laboratory for Oxo Synthesis and Selective Oxidation Department, Center for Excellence in Molecular Synthesis, Suzhou Research Institute of LICP, Lanzhou Institute of Chemical Physics (LICP), Chinese Academy of Sciences, Lanzhou 730000, P. R. China.

[b] University of Chinese Academy of Sciences, Beijing 100049, P. R. China.

Contents

1. Table S1. Energies of intermediates in the cyclohexane (CYH) hydroxylation by 1 and 2

2. Table S2. Mulliken charges and spins of the complex 1, 2, 2' and intermediates in the cyclohexane hydroxylation

3. Fig. S1 Key geometric parameters for 2'

4. Fig. S2 Natural d orbitals and their occupations in ^{3}TS and the spin natural orbitals and their occupations in ^{5}TS for the H-abstraction reaction of 2 with cyclohexane

5. Fig. S3. Spin density plot of (a) ^{3}1, (b) ^{5}1, (c) ^{3}2, (d) ^{5}2, and (e) ^{3}TS, (f) ^{5}TS for CYH +1; (g) ^{3}TS, (h) ^{5}TS for CYH +2

6. Fig. S4 Key geometric parameters of the transition state structures and energy profile for cyclohexane hydroxylation by 2'

7. Cartesian coordination

Table S1. Energies of intermediates in the cyclohexane (CYH) hydroxylation by **1** and **2**. Absolute energies are in a.u. units and the relative ones are in kcal mol^{-1}. [a]

	ZPE	cor to G	SPE	E	G	ΔE	ΔG
CYH	0.171036	0.147485	-235.997725	-235.826689	-235.85024	-	-
CYH radical	0.156117	0.131781	-235.330454	-235.174337	-235.198673	-	-
^{1}**1**	0.484606	0.444426	-2505.778097	-2505.293491	-2505.333671	26.4	26.8
^{3}**1**	0.484631	0.443849	-2505.820233	-2505.335602	-2505.376384	0.0	0.0
^{5}**1**	0.484016	0.440896	-2505.816643	-2505.332627	-2505.375747	1.9	0.4
^{1}**2**	0.543072	0.497636	-2622.544843	-2622.001771	-2622.047207	33.3	34.9
^{3}**2**	0.54323	0.495223	-2622.598054	-2622.054824	-2622.102831	0.0	0.0
^{5}**2**	0.542412	0.492284	-2622.592563	-2622.050151	-2622.100279	2.9	1.6
^{1}**2'**	0.542706	0.493997	-2622.551245	-2622.008539	-2622.057248	29.0	28.6
^{3}**2'**	0.543015	0.495165	-2622.597789	-2622.054774	-2622.102624	0.0 [b]	0.1
^{5}**2'**	0.542852	0.493396	-2622.593394	-2622.050542	-2622.099998	2.7	1.8
				CYH + **1**			
^{1}**RC**	0.656865	0.607569	-2741.785034	-2741.128169	-2741.177465	26.4	26.6
^{1}**TS**	0.650224	0.602839	-2741.772587	-2741.122363	-2741.169748	30.0	31.5
^{1}**PC**	0.660298	0.612418	-2741.878557	-2741.218259	-2741.266139	-30.1	-29.0
^{3}**RC**	0.657084	0.607437	-2741.827333	-2741.170249	-2741.219896	0.0	0.0
^{3}**TS**	0.6501	0.602418	-2741.798	-2741.1479	-2741.195582	14.0	15.3
^{3}**IM**	0.65415	0.6045	-2741.816013	-2741.161863	-2741.211513	5.3	5.3
^{3}**PC$_{reb}$**	0.656988	0.607172	-2741.877404	-2741.220416	-2741.270232	-31.5	-31.6
^{5}**RC**	0.656135	0.603533	-2741.824065	-2741.16793	-2741.220532	1.5	-0.4
^{5}**TS**	0.647033	0.596288	-2741.804786	-2741.157753	-2741.208498	7.8	7.2
^{5}**IM**	0.649575	0.598088	-2741.821161	-2741.171586	-2741.223073	-0.8	-2.0
^{5}**PC$_{reb}$**	0.655394	0.603843	-2741.893091	-2741.237697	-2741.289248	-42.3	-43.5
^{2}**FP$_{HAA}$**	0.496009	0.455594	-2506.474606	-2505.978597	-2506.019012	10.9	1.4
^{4}**FP$_{HAA}$**	0.495073	0.451899	-2506.460961	-2505.965888	-2506.009062	18.8	7.6
^{6}**FP$_{HAA}$**	0.491757	0.447694	-2506.475252	-2505.983495	-2506.027558	7.8	-4.0
				CYH + **2**			
^{1}**RC**	0.715272	0.715272	-2858.559574	-2857.844302	-2857.844302	28.5	64.1
^{1}**TS**	0.712953	0.660268	-2858.556401	-2857.843448	-2857.896133	29.0	31.5
^{1}**PC**	0.718438	0.666501	-2858.667902	-2857.949464	-2858.001401	-37.5	-34.5
^{3}**RC**	0.715328	0.658695	-2858.605069	-2857.889741	-2857.946374	0.0	0.0
^{3}**TS**	0.708582	0.654819	-2858.577685	-2857.869103	-2857.922866	13.0	14.8
^{3}**IM**	0.712525	0.657302	-2858.594381	-2857.881856	-2857.937079	4.9	5.8
^{3}**PC$_{reb}$**	0.715131	0.658086	-2858.662291	-2857.94716	-2858.004205	-36.0	-36.3
^{5}**RC**	0.714368	0.654951	-2858.600079	-2857.885711	-2857.945128	2.5	0.8
^{5}**TS**	0.706127	0.64908	-2858.580536	-2857.874409	-2857.931456	9.6	9.4
^{5}**IM**	0.707941	0.649587	-2858.596885	-2857.888944	-2857.947298	0.5	-0.6
^{5}**PC$_{reb}$**	0.713119	0.653128	-2858.675544	-2857.962425	-2858.022416	-45.6	-47.7
^{2}**FP$_{HAA}$**	0.553644	0.507338	-2623.251468	-2622.697824	-2622.74413	11.0	2.2
^{4}**FP$_{HAA}$**	0.552326	0.501724	-2623.245553	-2622.693227	-2622.743829	13.9	2.4

^{6}**FP$_{HAA}$**	0.549758	0.498694	-2623.249352	-2622.699594	-2622.750658	9.9	-1.9
			CYH + **2'**				
^{3}**RC**	0.716375	0.662029	-2858.606003	-2857.889628	-2857.943974	0.1	1.5
^{3}**TS**	0.709046	0.655957	-2858.578043	-2857.868997	-2857.922086	13.0	15.2
^{3}**IM**	0.712566	0.65717	-2858.595307	-2857.882741	-2857.938137	4.4	5.2
^{3}**PC$_{reb}$**	0.715272	0.658756	-2858.659536	-2857.944264	-2858.00078	-34.2	-34.1
^{5}**RC**	0.715045	0.656446	-2858.601472	-2857.886427	-2857.945026	2.1	0.8
^{5}**TS**	0.705868	0.648284	-2858.580472	-2857.874604	-2857.932188	9.5	8.9
^{5}**IM**	0.708158	0.650534	-2858.596786	-2857.888628	-2857.946252	0.7	0.1
^{5}**PC$_{reb}$**	0.713035	0.654633	-2858.676531	-2857.963496	-2858.021898	-46.3	-47.4
^{2}**FP$_{HAA}$**	0.554273	0.507387	-2623.252096	-2622.697823	-2622.744709	11.0	1.9
^{4}**FP$_{HAA}$**	0.552708	0.50242	-2623.245202	-2622.692494	-2622.742782	14.4	3.1
^{6}**FP$_{HAA}$**	0.549771	0.498817	-2623.249291	-2622.69952	-2622.750474	10.0	-1.7

[a] Energy of doublet CYH radical was included in the **FP$_{HAA}$**.

[b] The spin point energy of ^{3}**2'** is 0.03 kcal mol^{-1} higher than ^{3}**2**.

Table S2. Mulliken charges and spins of the complex **1**, **2** and intermediates in the cyclohexane hydroxylation at the B3LYP-D3(BJ)/B1 level. [a]

	Charge			Spin		
	Fe	O	L	Fe	O	L
^{1}**1**	0.60	-0.44	1.84	0.00	0.00	0.00
^{3}**1**	0.63	-0.43	1.80	1.25	0.83	-0.08
^{5}**1**	0.77	-0.44	1.67	3.14	0.62	0.24
^{1}**2**	0.59	-0.41	1.82	0.00	0.00	0.00
^{3}**2**	0.60	-0.41	1.81	1.22	0.87	-0.09
^{5}**2**	0.75	-0.43	1.67	3.14	0.66	0.19
^{1}**2'**	0.60	-0.42	1.82	0.00	0.00	0.00
^{3}**2'**	0.60	-0.41	1.81	1.22	0.87	-0.09
^{5}**2'**	0.75	-0.43	1.67	3.14	0.66	0.19

CYH + **1**

	Fe	O	L	H	CYH-H	Fe	O	L	H	CYH-H
^{3}**RC**	0.64	-0.44	1.79	0.16	-0.15	1.23	0.84	-0.07	0.00	0.00
^{3}**TS**	0.58	-0.60	1.65	0.34	0.02	0.94	0.58	-0.05	-0.05	0.57
^{3}**IM**	0.49	-0.70	1.69	0.46	0.05	0.94	0.16	-0.06	0.02	0.94
$^{3}\mathbf{PC_{reb}}$	0.56	-0.65	1.22	0.47	0.41	1.98	0.02	-0.01	0.00	0.01
^{5}**RC**	0.77	-0.45	1.67	0.16	-0.15	3.13	0.62	0.24	0.00	0.00
^{5}**TS**	0.84	-0.63	1.44	0.30	0.05	3.93	0.10	0.38	0.00	-0.42
^{5}**IM**	0.77	-0.76	1.44	0.45	0.11	4.15	0.31	0.46	-0.05	-0.87
$^{5}\mathbf{PC_{reb}}$	0.68	-0.66	1.09	0.47	0.42	3.78	0.02	0.20	0.00	0.01

CYH + **2**

	Fe	O	L	CH_3CN	H	CYH-H	Fe	O	L	CH_3CN	H	CYH-H
^{3}**RC**	0.61	-0.41	1.53	0.26	0.17	-0.15	1.20	0.88	-0.07	-0.01	0.00	0.01
^{3}**TS**	0.64	-0.56	1.32	0.33	0.18	0.10	0.94	0.59	-0.07	-0.01	-0.01	0.55
^{3}**IM**	0.47	-0.68	1.43	0.25	0.47	0.06	0.95	0.18	-0.07	-0.01	0.02	0.93
$^{3}\mathbf{PC_{reb}}$	0.54	-0.64	1.05	0.20	0.46	0.39	1.99	0.02	0.01	-0.03	0.00	0.01
^{5}**RC**	0.74	-0.43	1.44	0.23	0.17	-0.15	3.15	0.66	0.15	0.04	0.00	0.00
^{5}**TS**	0.81	-0.60	1.22	0.22	0.30	0.05	3.92	0.11	0.30	0.04	0.00	-0.38
^{5}**IM**	0.76	-0.75	1.18	0.22	0.45	0.13	4.14	0.33	0.38	0.05	-0.05	-0.85
$^{5}\mathbf{PC_{reb}}$	0.66	-0.67	0.93	0.19	0.47	0.42	3.78	0.03	0.17	0.02	0.00	0.01

CYH + **2'**

	Fe	O	L	CH_3CN	H	CYH-H	Fe	O	L	CH_3CN	H	CYH-H
^{3}**RC**	0.61	-0.42	1.53	0.26	0.17	-0.15	1.20	0.88	-0.07	-0.01	0.00	0.01
^{3}**TS**	0.55	-0.58	1.40	0.24	0.35	0.03	0.93	0.62	-0.06	-0.01	-0.05	0.57
^{3}**IM**	0.47	-0.68	1.43	0.25	0.47	0.06	0.95	0.18	-0.07	-0.01	0.02	0.93
$^{3}\mathbf{PC_{reb}}$	0.53	-0.65	1.05	0.20	0.47	0.40	1.99	0.02	-0.03	0.01	0.00	0.01
^{5}**RC**	0.76	-0.43	1.42	0.22	0.14	-0.12	3.13	0.67	0.15	0.04	0.00	0.01
^{5}**TS**	0.82	-0.61	1.22	0.22	0.30	0.05	3.93	0.11	0.30	0.05	0.00	-0.39
^{5}**IM**	0.77	-0.75	1.19	0.22	0.45	0.13	4.15	0.32	0.38	0.05	-0.05	-0.85
$^{5}\mathbf{PC_{reb}}$	0.66	-0.66	0.92	0.19	0.47	0.41	3.78	0.02	0.17	0.02	0.00	0.01

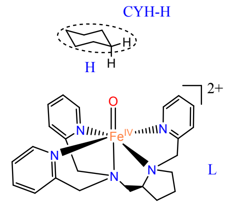	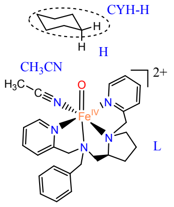
**1**	**2**

[a] CYH-H is the CYH radical.

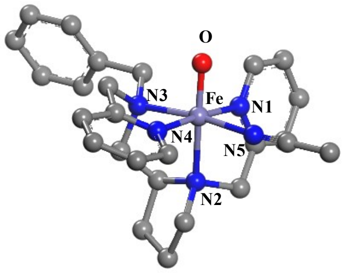

Fe-O	1.622/1.629/1.635
Fe-N1	2.051/2.004/2.013
Fe-N2	2.157/2.136/2.130
Fe-N3	2.223/2.032/2.046
Fe-N4	2.107/1.986/1.980
Fe-N5	2.217/1.974/1.963
∠N2-Fe-O	170.0/170.9/166.5

$^{5/3/1}$**2'**

ΔE 2.7/0.0/29.0

Fig. S1 Key geometric parameters for **2'** at the UB3LYP-D3(BJ)/B1 level and spin-state energy gaps at the UB3LYP-D3(BJ)/B2//B1 level (including ZPE and solvationcorrections).

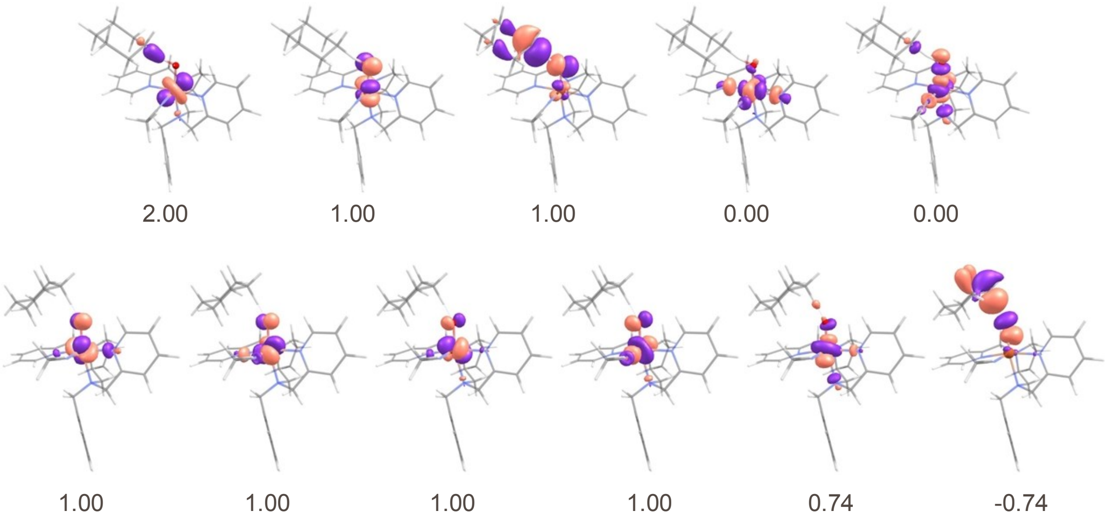

Fig. S2 Natural d orbitals and their occupations in ^{3}**TS** (top panel) and the spin natural orbitals and their occupations in ^{5}**TS** (bottom panel) for the H-abstraction reaction of **2** with cyclohexane.

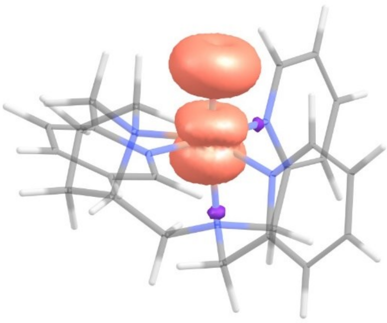

^{3}**1**

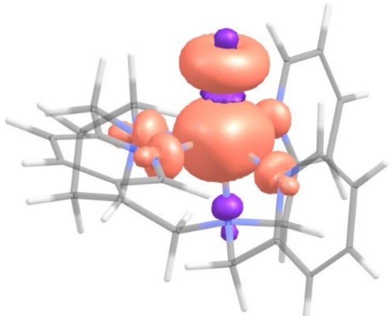

^{5}**1**

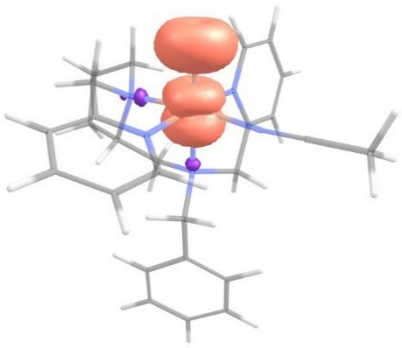

^{3}**2**

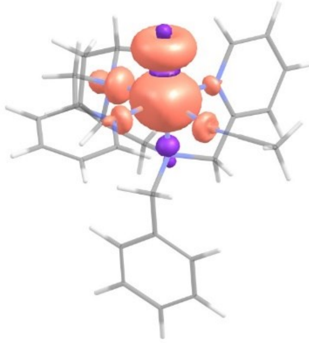

^{5}**2**

Fig. S3 Spin density plot of ^{3}**1**, ^{5}**1**, ^{3}**2**, and ^{5}**2**, contour value = 0.005.

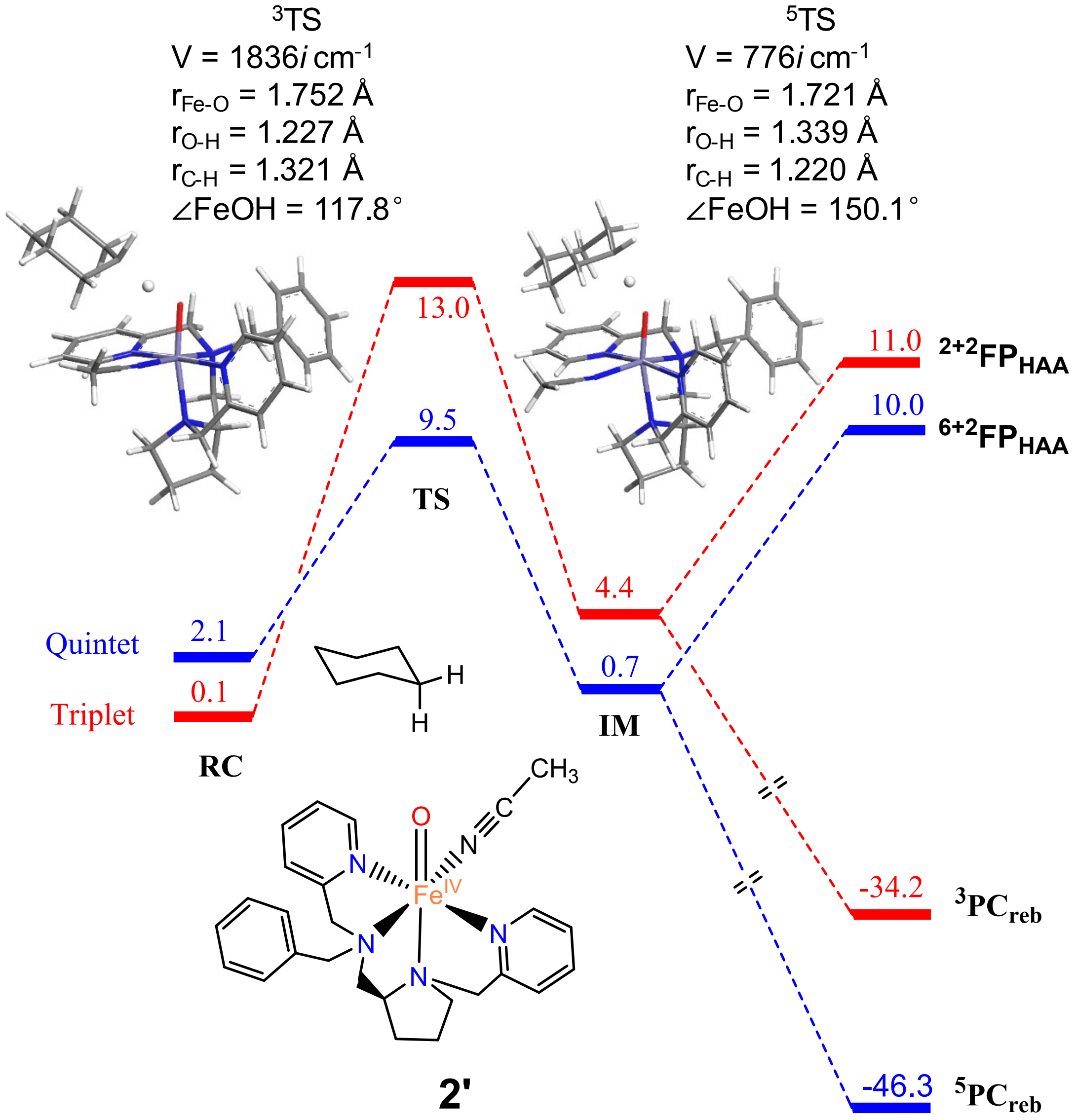

Fig. S4 Key geometric parameters of the transition state structures and energy profile for cyclohexane hydroxylation by **2'** at the UB3LYP-D3(BJ)/B2//B1 level with solvent correction. **FR**: free reactants, **RC**: reactant cluster, **TS**: transition state, **IM**: intermediate, **PC**$_{\mathbf{reb}}$: rebound productions, **FP**: free productions after H-atom abstraction.

Cartesian coordinates

^{1}1

Fe 7.72827 7.94766 3.08533
O 9.07383 8.65178 2.49592
N 6.67987 7.59163 1.45953
N 5.8369 7.30263 3.86091
N 6.87235 9.72302 3.413
N 8.32274 7.59104 4.96499
N 8.69752 6.15841 2.80552
C 6.97138 8.14002 0.26538
H 7.75814 8.88292 0.2577
C 6.29583 7.75343 -0.88552
H 6.55324 8.21118 -1.83303
C 5.30064 6.78145 -0.79144
H 4.75897 6.46156 -1.67471
C 4.99494 6.23202 0.45484
H 4.21308 5.49012 0.56828
C 5.70425 6.65636 1.56955
C 5.46703 6.17261 2.97638
H 4.42779 5.86112 3.12442
H 6.11118 5.31989 3.2092
C 4.86168 8.41992 3.69915
H 4.06565 8.34518 4.44503
H 4.38378 8.3369 2.71959
C 5.5721 9.74018 3.77142
C 4.93061 10.92337 4.12256
H 3.8883 10.90196 4.42013
C 5.64686 12.11804 4.07942
H 5.16793 13.05242 4.35184
C 6.98424 12.09311 3.68405
H 7.57683 12.99896 3.63459
C 7.56639 10.87451 3.36136
H 8.60026 10.77566 3.05771
C 5.9757 6.94856 5.29815
H 5.29095 6.13893 5.56899
H 5.68556 7.82464 5.88089
C 7.40978 6.57087 5.62252
H 7.64805 5.59111 5.20876
C 7.71396 6.67374 7.13216
H 6.78821 6.58576 7.70624
H 8.36908 5.86166 7.45192
C 8.3912 8.05888 7.32825
H 9.42068 7.94076 7.67386
H 7.86801 8.67467 8.06196
C 8.35122 8.72834 5.94483
H 7.4402 9.3098 5.81605
H 9.20207 9.37204 5.72197
C 9.68877 6.99802 4.8239
H 10.02332 6.56613 5.77046
H 10.3715 7.80627 4.55083
C 9.65136 5.96459 3.74061
C 10.53872 4.89698 3.68248
H 11.27765 4.76828 4.46516
C 10.4571 4.01194 2.60935
H 11.13933 3.17184 2.53783
C 9.48371 4.22364 1.63479
H 9.38208 3.56386 0.78139
C 8.62365 5.30676 1.76749
H 7.86247 5.49617 1.02448

^{3}1

Fe 7.71339 7.92841 3.07761
O 9.0269 8.65981 2.45641
N 6.69584 7.61103 1.40373
N 5.84304 7.2804 3.83443
N 6.85236 9.69547 3.42992
N 8.35894 7.63192 4.97417
N 8.66536 6.14072 2.84187
C 6.99069 8.15372 0.21246
H 7.7933 8.88083 0.20929
C 6.30747 7.77932 -0.93938
H 6.5669 8.23383 -1.88797
C 5.30055 6.82006 -0.84286
H 4.75159 6.50846 -1.72474
C 4.99368 6.26851 0.40316
H 4.20653 5.53179 0.51507
C 5.71212 6.68262 1.51612
C 5.47702 6.1705 2.92042
H 4.43743 5.85695 3.06016
H 6.11666 5.31036 3.13625
C 4.85173 8.38752 3.69052
H 4.05659 8.28872 4.43436
H 4.37676 8.3158 2.70868
C 5.55121 9.709 3.78443
C 4.91044 10.88851 4.14821
H 3.86707 10.86419 4.44148
C 5.62848 12.08226 4.12336
H 5.14995 13.01387 4.40541
C 6.96754 12.06089 3.73265
H 7.56189 12.96606 3.69655
C 7.5492 10.84686 3.39497
H 8.58565 10.75768 3.09582
C 6.0017 6.92805 5.27416
H 5.34604 6.09461 5.54461
H 5.67611 7.79452 5.85288
C 7.4476 6.60281 5.62457
H 7.71374 5.62149 5.23334
C 7.71603 6.71946 7.1429
H 6.77282 6.67658 7.69368
H 8.33069 5.88787 7.49187
C 8.43852 8.08028 7.33916
H 9.47105 7.92733 7.66234
H 7.95025 8.70427 8.09006
C 8.39001 8.75735 5.9613
H 7.47364 9.33458 5.84407
H 9.23586 9.40906 5.74089
C 9.71759 7.04139 4.81026
H 10.0944 6.63794 5.75397
H 10.38676 7.84159 4.48362
C 9.6461 5.97593 3.75854
C 10.52886 4.90487 3.70279
H 11.28951 4.79756 4.46747
C 10.41664 3.98929 2.65848
H 11.09584 3.14632 2.5928
C 9.41819 4.17475 1.70418
H 9.29302 3.4917 0.87264
C 8.56379 5.26248 1.8272
H 7.7859 5.43517 1.09808

^{5}1

Fe 7.73429 8.13075 3.00741
O 9.05944 8.84336 2.41256
N 6.67752 7.57356 1.27858
N 5.8613 7.44194 3.78887
N 6.80673 9.93835 3.48453
N 8.42887 7.59193 4.98793
N 8.7574 6.09545 2.76676
C 6.96835 7.97585 0.03015
H 7.76549 8.7043 -0.06187
C 6.28705 7.46959 -1.07013
H 6.54229 7.81046 -2.06632
C 5.28178 6.52546 -0.85877

H 4.73105 6.11339 -1.69738
C 4.98051 6.11749 0.44236
H 4.19721 5.39403 0.63685
C 5.70291 6.65925 1.49754
C 5.49014 6.27695 2.94364
H 4.45444 5.97329 3.12741
H 6.13697 5.43684 3.2109
C 4.85311 8.53302 3.61911
H 4.01066 8.37877 4.29958
H 4.45954 8.48575 2.59993
C 5.49936 9.87357 3.81922
C 4.81202 11.00107 4.25225
H 3.76643 10.92391 4.52703
C 5.49121 12.21753 4.32042
H 4.97478 13.10985 4.65744
C 6.83708 12.27562 3.95438
H 7.39417 13.20398 3.99331
C 7.46678 11.10981 3.54151
H 8.51089 11.07728 3.25372
C 6.01865 7.12087 5.23917
H 5.27561 6.3759 5.54079
H 5.80176 8.03691 5.7925
C 7.42243 6.63704 5.58149
H 7.59374 5.6484 5.15557
C 7.6737 6.67419 7.1073
H 6.72473 6.6451 7.64895
H 8.25021 5.80218 7.42136
C 8.45472 7.99178 7.36654
H 9.45818 7.77831 7.74231
H 7.9589 8.63029 8.10026
C 8.5228 8.69235 5.99795
H 7.67124 9.35766 5.85628
H 9.43244 9.26802 5.82435
C 9.74344 6.9263 4.81723
H 10.08243 6.47332 5.75411
H 10.46573 7.69859 4.5366
C 9.67258 5.88741 3.73064
C 10.52942 4.7902 3.70569
H 11.24134 4.64485 4.51055
C 10.45208 3.89866 2.63826
H 11.10984 3.03714 2.59529
C 9.51558 4.12934 1.63089
H 9.41948 3.46298 0.78177
C 8.68718 5.2404 1.73277
H 7.94895 5.4527 0.97086

^{1}2

Fe -0.01276 0.06429 0.0312
O 0.02658 -0.06172 1.66011
N 2.00824 0.06976 0.01097
N 0.19717 -0.2029 -2.09812
N 0.2678 2.03137 0.02106
N -0.33142 -1.91015 -0.1516
C 1.10535 1.20417 -5.07271
H 1.95593 1.60073 -4.52444
C 1.19172 1.04512 -6.45638
H 2.11149 1.30313 -6.97231
C 0.09437 0.56501 -7.17391
H 0.15933 0.44296 -8.25087
C -1.09248 0.25662 -6.50482
H -1.95489 -0.10028 -7.05953
C -1.1763 0.41588 -5.12176
H -2.10885 0.19141 -4.61146
C -0.0742 0.87734 -4.38888
C -0.15609 1.03331 -2.88962
H 0.51328 1.82615 -2.55818
H -1.17007 1.30489 -2.58995
C 1.61446 -0.61472 -2.29995
H 1.90124 -0.5107 -3.3495
H 1.69482 -1.67455 -2.05025
C 2.53148 0.19062 -1.40291
H 2.50536 1.24526 -1.68057
C 3.97479 -0.37008 -1.37321
H 4.11055 -1.08217 -2.19172
H 4.70734 0.42689 -1.51188
C 4.12934 -1.05692 0.00818
H 4.58037 -2.04815 -0.06821
H 4.7583 -0.45975 0.67348
C 2.6994 -1.14828 0.5493
H 2.19129 -2.0261 0.15258
H 2.61227 -1.16211 1.63552
C 2.40585 1.27348 0.7976
H 3.45535 1.52402 0.62613
H 2.27171 1.03736 1.85502
C 1.50248 2.40559 0.42428
C 1.86076 3.74523 0.51217
H 2.86368 4.01297 0.82386
C 0.91509 4.71972 0.19849
H 1.17308 5.77136 0.25941
C -0.36307 4.32455 -0.19546
H -1.12924 5.04821 -0.446
C -0.65222 2.9696 -0.27275
H -1.6276 2.61887 -0.57896
C -0.90324 -3.71795 -1.60282
H -1.12567 -4.06147 -2.60647
C -0.88312 -4.59753 -0.52234
H -1.09142 -5.65146 -0.67147
C -0.59157 -4.10451 0.74974
H -0.56946 -4.75263 1.6177
C -0.32133 -2.75152 0.89926
H -0.09832 -2.30225 1.8586
C -0.63244 -2.37213 -1.38269
C -0.69406 -1.3317 -2.46209
H -0.42837 -1.75178 -3.4363
H -1.71578 -0.95073 -2.53666
N -1.96925 0.21296 -0.14479
C -3.12361 0.21784 -0.14493
C -4.57402 0.23004 -0.15617
H -4.92947 0.2883 -1.18897
H -4.94872 -0.68733 0.30691
H -4.93631 1.09651 0.40425

^{3}2

Fe 0.01921 0.08014 0.08028
O -0.01185 0.02839 1.70638
N 2.02753 0.06127 0.03881
N 0.1798 -0.17647 -2.06222
N 0.30975 2.04926 0.05464
N -0.27581 -1.89273 -0.09068
C 1.03574 1.1937 -5.07138
H 1.89428 1.60028 -4.5431
C 1.09865 1.01675 -6.45419
H 2.0084 1.27099 -6.98944
C -0.0093 0.52374 -7.14619
H 0.03732 0.38786 -8.22246
C -1.18328 0.2204 -6.45257
H -2.054 -0.14644 -6.98749
C -1.24357 0.39771 -5.07043
H -2.16644 0.1776 -4.54095
C -0.13045 0.87192 -4.36275
C -0.18719 1.0461 -2.86427
H 0.48756 1.84354 -2.55405

H -1.19577 1.32194 -2.5507
C 1.59592 -0.59184 -2.27967
H 1.87297 -0.47791 -3.3307
H 1.67631 -1.6545 -2.04279
C 2.52884 0.19855 -1.38547
H 2.50047 1.25709 -1.64757
C 3.97155 -0.36446 -1.38381
H 4.0895 -1.07662 -2.20504
H 4.70254 0.43127 -1.53661
C 4.15297 -1.05374 -0.00717
H 4.60964 -2.04151 -0.094
H 4.788 -0.45492 0.65076
C 2.73205 -1.15897 0.55405
H 2.2244 -2.03445 0.15298
H 2.66037 -1.18575 1.64115
C 2.43739 1.2618 0.82801
H 3.49024 1.49976 0.66001
H 2.29994 1.02363 1.88602
C 1.54997 2.4078 0.45333
C 1.92981 3.74178 0.53276
H 2.93776 3.99638 0.83929
C 0.99707 4.72789 0.21586
H 1.26992 5.77608 0.27109
C -0.2873 4.34905 -0.17399
H -1.0432 5.08281 -0.4265
C -0.59673 2.99778 -0.24408
H -1.57743 2.65873 -0.54626
C -0.8845 -3.70223 -1.52407
H -1.13525 -4.04833 -2.52014
C -0.82903 -4.5807 -0.44382
H -1.038 -5.63542 -0.58622
C -0.50076 -4.08684 0.81912
H -0.45003 -4.73506 1.68573
C -0.22986 -2.73295 0.95952
H 0.02986 -2.28422 1.90986
C -0.61338 -2.3548 -1.31335
C -0.71289 -1.31642 -2.3937
H -0.47053 -1.73834 -3.37326
H -1.73936 -0.9443 -2.44218
C -3.1008 0.27692 -0.07699
C -4.55117 0.31121 -0.06362
H -4.94146 -0.57557 -0.57115
H -4.90469 0.32402 0.97132
H -4.90037 1.2105 -0.57891
N -1.94679 0.25565 -0.09538

^{5}2

Fe -0.00349 0.05656 0.11607
O -0.018 0.00016 1.73589
N 2.17128 0.13279 0.00435
N 0.20281 -0.20227 -2.04176
N 0.34015 2.14541 0.06372
N -0.40244 -1.9482 -0.09814
C 1.18394 1.20674 -4.98383
H 2.03005 1.58366 -4.41521
C 1.29547 1.05366 -6.36613
H 2.22964 1.29826 -6.86231
C 0.20437 0.59578 -7.10757
H 0.28859 0.47808 -8.18367
C -1.00021 0.30319 -6.46366
H -1.85688 -0.03721 -7.03726
C -1.10917 0.45659 -5.08151
H -2.05493 0.24387 -4.591
C -0.01436 0.89702 -4.32538
C -0.1183 1.04657 -2.82735
H 0.56142 1.82284 -2.47924
H -1.12957 1.33443 -2.53467
C 1.6121 -0.65683 -2.24783
H 1.86241 -0.62379 -3.31161
H 1.67072 -1.70222 -1.93794
C 2.59938 0.16802 -1.43829
H 2.58992 1.20777 -1.7695
C 4.02573 -0.44199 -1.47675
H 4.0791 -1.2146 -2.24887
H 4.76874 0.31757 -1.72535
C 4.26281 -1.03458 -0.06468
H 4.70139 -2.03391 -0.09937
H 4.93604 -0.39761 0.51571
C 2.86933 -1.0607 0.57362
H 2.3164 -1.95231 0.27068
H 2.85626 -1.00745 1.66233
C 2.55095 1.37829 0.71091
H 3.5842 1.66337 0.49104
H 2.47482 1.18543 1.78509
C 1.61027 2.49776 0.34889
C 2.00433 3.83192 0.35129
H 3.03686 4.0852 0.56393
C 1.05774 4.81866 0.08227
H 1.34441 5.86475 0.08064
C -0.25848 4.44349 -0.18684
H -1.02607 5.17781 -0.39988
C -0.57997 3.09296 -0.18976
H -1.58295 2.74767 -0.40316
C -0.98463 -3.70877 -1.59833
H -1.20363 -4.03118 -2.60963
C -0.98619 -4.61031 -0.5341
H -1.20968 -5.65718 -0.709
C -0.69349 -4.15587 0.75283
H -0.68452 -4.82782 1.60249
C -0.4043 -2.81141 0.93542
H -0.16772 -2.38827 1.90422
C -0.69396 -2.37344 -1.34831
C -0.72447 -1.30837 -2.40744
H -0.47456 -1.71917 -3.38961
H -1.73487 -0.89513 -2.46827
C -3.33428 0.38117 -0.1861
C -4.78512 0.35319 -0.15163
H -5.17439 0.27338 -1.17054
H -5.11873 -0.5081 0.43431
H -5.15748 1.27238 0.30963
N -2.17813 0.40719 -0.22078

^{1}2'

Fe -0.00263 -0.00715 -0.00056
O -0.01222 -0.01046 1.63427
N 0.50827 -0.00111 -2.06886
N 1.03343 1.757 0.04273
N 1.55372 -1.28318 -0.01472
N -1.5333 1.23972 -0.15446
C 4.10849 3.2496 -0.06858
H 4.31158 2.57482 -0.89623
C 4.82666 4.44078 0.04093
H 5.57247 4.6915 -0.70713
C 4.59132 5.30127 1.11517
H 5.1509 6.22762 1.20314
C 3.64475 4.96068 2.08428
H 3.46963 5.61765 2.93075
C 2.92804 3.76933 1.97298
H 2.20714 3.50319 2.74059
C 3.14179 2.90794 0.88791
C 2.36081 1.6241 0.756
H 2.94082 0.88546 0.20432

H 2.12602 1.20546 1.73561
C 1.19543 2.22737 -1.36633
H 1.95552 3.01067 -1.41085
H 0.25252 2.67747 -1.68167
C 1.54529 1.07288 -2.2819
H 2.51839 0.65785 -2.01414
C 1.47062 1.46688 -3.77919
H 1.40301 2.5542 -3.87422
H 2.36786 1.15099 -4.31409
C 0.19904 0.77194 -4.32674
H -0.41078 1.43973 -4.93866
H 0.45936 -0.09288 -4.94276
C -0.55881 0.33424 -3.0684
H -1.14909 1.15938 -2.6714
H -1.21765 -0.52403 -3.20474
C 1.10186 -1.32842 -2.37662
H 1.69781 -1.30351 -3.29332
H 0.286 -2.03954 -2.53419
C 1.9329 -1.77406 -1.21178
C 2.99155 -2.66874 -1.32424
H 3.28286 -3.0324 -2.30289
C 3.65312 -3.08182 -0.16986
H 4.48221 -3.77818 -0.23449
C 3.23245 -2.59067 1.06639
H 3.71275 -2.89219 1.98964
C 2.17571 -1.69237 1.10688
H 1.79293 -1.28271 2.03281
C -2.24769 3.47146 0.31171
H -2.00146 4.46793 0.65983
C -3.53471 3.15197 -0.11812
H -4.31583 3.90435 -0.1091
C -3.80213 1.85804 -0.56465
H -4.78709 1.56894 -0.9109
C -2.77486 0.92489 -0.56947
H -2.93148 -0.08802 -0.91373
C -1.26631 2.48823 0.29031
C 0.13647 2.69813 0.77214
H 0.46463 3.73088 0.63185
H 0.19026 2.45457 1.83467
C -1.69895 -2.60191 -0.28654
N -1.10191 -1.6154 -0.23962
C -2.45004 -3.84143 -0.3545
H -1.75944 -4.68822 -0.30705
H -3.14778 -3.8914 0.48623
H -3.00848 -3.87817 -1.29427

[3]2'

Fe 17.46421 0.0968 17.99378
O 18.52572 -1.13794 17.96438
N 16.31879 1.89663 17.88975
N 18.53281 1.0835 19.41354
N 18.28693 0.96255 16.38425
N 16.54415 -0.6295 19.59706
C 20.81299 3.52476 20.12666
H 20.22824 4.14605 19.45318
C 21.60528 4.12687 21.10464
H 21.62054 5.20859 21.19555
C 22.3824 3.33871 21.95579
H 23.00117 3.80568 22.71606
C 22.37391 1.94881 21.81698
H 22.98962 1.33194 22.46434
C 21.5813 1.34883 20.83861
H 21.59561 0.26864 20.72546
C 20.78067 2.12927 19.99304
C 19.91223 1.48619 18.94001
H 19.76931 2.16894 18.10363
H 20.36842 0.57232 18.5583
C 17.72059 2.26254 19.85253
H 18.35032 2.95296 20.41798
H 16.94681 1.90232 20.5324
C 17.07905 2.94728 18.66388
H 17.84629 3.3606 18.00698
C 16.04065 4.01664 19.09153
H 16.11667 4.19811 20.16734
H 16.22582 4.96711 18.58855
C 14.65901 3.42497 18.7184
H 13.91828 3.56469 19.50831
H 14.26519 3.89151 17.8113
C 14.94432 1.93827 18.48372
H 14.96752 1.39832 19.43055
H 14.24165 1.43451 17.81894
C 16.27285 2.28422 16.4543
H 16.0248 3.34236 16.33159
H 15.48193 1.70417 15.97095
C 17.59041 1.96611 15.8112
C 18.06134 2.61287 14.6734
H 17.48932 3.42791 14.24512
C 19.2612 2.19179 14.10395
H 19.64676 2.68181 13.21644
C 19.95592 1.13176 14.68737
H 20.88739 0.76655 14.27161
C 19.4369 0.53825 15.82906
H 19.92292 -0.29108 16.32656
C 16.82924 -1.04923 21.93227
H 17.44043 -0.95114 22.82181
C 15.58449 -1.67579 21.97303
H 15.20718 -2.08038 22.90589
C 14.83031 -1.76956 20.80365
H 13.85712 -2.24534 20.79293
C 15.3422 -1.23295 19.63
H 14.79233 -1.27451 18.70016
C 17.28705 -0.54258 20.7226
C 18.62469 0.11033 20.54182
H 18.95981 0.60947 21.45435
H 19.36325 -0.64726 20.26802
C 15.55976 -1.28954 15.94107
N 16.20474 -0.75531 16.73503
C 14.73923 -1.95485 14.94611
H 14.63102 -1.30712 14.07148
H 15.21709 -2.89259 14.64851
H 13.75261 -2.16624 15.36814

[5]2'

Fe 0.54625 0.02926 0.63303
O 1.01757 0.05099 2.18514
N 0.28257 0.05347 -1.5075
N 1.23518 2.10837 0.25168
N 2.19906 -1.02093 0.02444
N -1.18451 1.15727 1.04879
C 3.78554 4.01314 -0.96399
H 3.80163 3.31969 -1.80081
C 4.28133 5.3069 -1.13153
H 4.66572 5.61618 -2.09872
C 4.29014 6.19598 -0.05499
H 4.67733 7.20232 -0.18314
C 3.81225 5.78326 1.19115
H 3.83152 6.46495 2.03605
C 3.31731 4.4897 1.35641
H 2.96362 4.17096 2.33293
C 3.28702 3.5951 0.27761
C 2.7232 2.20474 0.44566
H 3.18333 1.52145 -0.2699

H 2.92125 1.818 1.44729
C 0.79566 2.44176 -1.12831
H 1.33447 3.31913 -1.49847
H -0.26202 2.7105 -1.08991
C 0.9912 1.26684 -2.07401
H 2.0517 1.02383 -2.15657
C 0.34263 1.52832 -3.45758
H 0.09329 2.58838 -3.55414
H 1.03331 1.28423 -4.26654
C -0.92747 0.64083 -3.50019
H -1.80672 1.18823 -3.84545
H -0.78694 -0.21164 -4.16987
C -1.10909 0.16921 -2.05294
H -1.63858 0.91884 -1.46552
H -1.62733 -0.78366 -1.9405
C 0.93215 -1.18811 -2.02024
H 1.13245 -1.11905 -3.09245
H 0.23629 -2.01737 -1.86613
C 2.19411 -1.45657 -1.25539
C 3.28167 -2.14345 -1.78032
H 3.26377 -2.47049 -2.81348
C 4.37975 -2.39746 -0.95873
H 5.23947 -2.93064 -1.35001
C 4.36386 -1.95783 0.36614
H 5.19811 -2.13904 1.03304
C 3.25211 -1.268 0.8267
H 3.17178 -0.89428 1.84027
C -1.98314 3.32335 1.64896
H -1.76846 4.36942 1.83569
C -3.28144 2.82432 1.73878
H -4.1023 3.48242 2.00251
C -3.51148 1.47344 1.47899
H -4.50524 1.04541 1.53368
C -2.43446 0.66799 1.13393
H -2.55646 -0.38449 0.91367
C -0.94692 2.45903 1.3119
C 0.49163 2.90684 1.25107
H 0.55286 3.97649 1.02906
H 0.95707 2.73589 2.22642
C -1.04577 -2.92342 0.79283
N -0.61742 -1.85816 0.65234
C -1.5923 -4.25734 0.96384
H -0.94907 -4.98261 0.45735
H -1.64246 -4.49725 2.02972
H -2.59676 -4.29891 0.53297

CYH + **1**

[3]**RC**

Fe -0.30067 0.48726 -0.35586
O -0.55543 0.80419 1.21827
N 1.68138 0.45899 -0.3685
N 0.10359 0.41717 -2.43203
N -0.4244 2.4322 -0.79423
N -2.19415 0.03732 -0.90308
N -0.32786 -1.506 0.08322
C 2.45814 0.87245 0.64431
H 1.94306 1.32822 1.48017
C 3.8374 0.69817 0.61327
H 4.43868 1.03635 1.44848
C 4.41251 0.08181 -0.49666
H 5.48536 -0.06805 -0.548
C 3.59686 -0.33442 -1.55191
H 4.01485 -0.80267 -2.43559
C 2.22613 -0.13643 -1.46026
C 1.23248 -0.54088 -2.52856
H 1.68991 -0.52631 -3.52327
H 0.8529 -1.5507 -2.35095
C 0.56351 1.76505 -2.87947
H 0.36558 1.90386 -3.94563
H 1.64651 1.83282 -2.74632
C -0.081 2.82365 -2.03851
C -0.26042 4.13596 -2.46228
H 0.00938 4.41441 -3.47453
C -0.78209 5.06851 -1.56789
H -0.93224 6.09743 -1.87646
C -1.10682 4.66008 -0.27416
H -1.51033 5.35331 0.45422
C -0.91919 3.3305 0.07771
H -1.16532 2.94428 1.05852
C -1.14812 0.04322 -3.15114
H -0.92314 -0.56683 -4.03135
H -1.60061 0.97016 -3.50896
C -2.13339 -0.68101 -2.24209
H -1.79253 -1.70004 -2.06376
C -3.57645 -0.63617 -2.79609
H -3.56081 -0.4032 -3.86394
H -4.06291 -1.60718 -2.68555
C -4.3075 0.46155 -1.97564
H -5.08221 0.02128 -1.34343
H -4.78995 1.20432 -2.61375
C -3.21032 1.11458 -1.12203
H -2.7283 1.92671 -1.66472
H -3.54521 1.49839 -0.15799
C -2.66027 -0.91709 0.14326
H -3.596 -1.40137 -0.14827
H -2.83538 -0.34496 1.05791
C -1.57934 -1.92956 0.37247
C -1.82727 -3.20812 0.85686
H -2.84604 -3.51225 1.06748
C -0.75555 -4.07488 1.05999
H -0.92383 -5.07728 1.43783
C 0.53304 -3.63104 0.76916
H 1.39874 -4.2667 0.90975
C 0.70811 -2.33872 0.29192
H 1.69722 -1.96343 0.07573
C 1.38492 -3.20287 4.4427
H 1.57138 -4.07082 5.08766
H 1.38174 -3.5747 3.40914
C 2.51283 -2.17247 4.59665
H 2.5915 -1.87672 5.65311
H 3.47601 -2.62075 4.32186
C 0.01202 -2.59195 4.75974
H -0.78014 -3.33062 4.58189
H -0.02872 -2.33639 5.82874
C -0.24918 -1.32569 3.93166
H -1.21024 -0.87766 4.21461
H -0.3327 -1.59576 2.87185
C 2.24303 -0.92293 3.74659
H 2.25245 -1.20536 2.68375
H 3.04603 -0.18696 3.88172
C 0.88243 -0.30167 4.08996
H 0.90568 0.05748 5.12944
H 0.68417 0.56787 3.45332

[5]**RC**

Fe -0.33692 0.19538 0.14363
O -0.58507 0.42865 1.72453
N 1.74405 0.04225 0.01392
N -0.00865 0.1551 -1.9653
N -0.64801 2.20316 -0.34705
N -2.26213 -0.58798 -0.40668
N -0.22193 -2.09617 0.49903

C 2.60328 0.3215 1.00849
H 2.17023 0.73952 1.90912
C 3.96341 0.06731 0.88153
H 4.6328 0.29587 1.702
C 4.4326 -0.48564 -0.31041
H 5.48896 -0.69461 -0.43963
C 3.53437 -0.76511 -1.34268
H 3.87225 -1.18799 -2.28171
C 2.18593 -0.49439 -1.14866
C 1.1147 -0.79592 -2.17152
H 1.50559 -0.72448 -3.1916
H 0.73567 -1.81133 -2.02971
C 0.40299 1.5234 -2.40506
H 0.23957 1.64005 -3.48023
H 1.47597 1.63442 -2.22505
C -0.32425 2.56703 -1.60715
C -0.59815 3.84507 -2.07923
H -0.34328 4.1111 -3.09863
C -1.19845 4.76438 -1.21862
H -1.42279 5.76727 -1.56577
C -1.50821 4.38347 0.08819
H -1.97161 5.07319 0.78337
C -1.22068 3.08671 0.49087
H -1.4459 2.71708 1.48427
C -1.27943 -0.23722 -2.64714
H -1.05829 -0.70369 -3.6122
H -1.82733 0.68506 -2.85086
C -2.13907 -1.16627 -1.79657
H -1.66992 -2.14678 -1.71989
C -3.58635 -1.2455 -2.33577
H -3.61287 -0.96931 -3.39293
H -3.96718 -2.26569 -2.26058
C -4.41168 -0.26707 -1.45566
H -5.166 -0.80826 -0.87967
H -4.93189 0.48659 -2.05008
C -3.38438 0.39696 -0.52141
H -2.99033 1.31323 -0.96101
H -3.76043 0.63579 0.47385
C -2.599 -1.64596 0.57919
H -3.49789 -2.19246 0.27803
H -2.80935 -1.14833 1.5303
C -1.44426 -2.59505 0.75485
C -1.62852 -3.90654 1.18704
H -2.63035 -4.27705 1.37339
C -0.51177 -4.71852 1.37042
H -0.62781 -5.74276 1.70791
C 0.75475 -4.19501 1.11052
H 1.65198 -4.78937 1.23583
C 0.85523 -2.87698 0.6817
H 1.82135 -2.43482 0.47752
C 1.41578 -3.80158 4.85049
H 1.59675 -4.65577 5.51514
H 1.39269 -4.19894 3.82671
C 2.56161 -2.78583 4.96613
H 2.65688 -2.46626 6.01428
H 3.51449 -3.25594 4.69188
C 0.05727 -3.16001 5.1688
H -0.74893 -3.89047 5.0236
H 0.03568 -2.87221 6.23018
C -0.19404 -1.91514 4.30609
H -1.14551 -1.44431 4.58445
H -0.29169 -2.21731 3.25624
C 2.30362 -1.55193 4.0895
H 2.30361 -1.85753 3.03334
H 3.11747 -0.82454 4.20525
C 0.95378 -0.90369 4.4259
H 0.99003 -0.51603 5.45474
H 0.76194 -0.04864 3.76764

^{3}TS

Fe -0.06393 0.13225 0.2899
O -0.19673 0.4812 2.00258
N 1.91502 0.07597 0.12397
N 0.18984 0.13723 -1.78976
N -0.22716 2.09545 -0.05607
N -2.00683 -0.29596 -0.14101
N -0.07493 -1.87362 0.69598
C 2.77906 0.47634 1.06875
H 2.33773 0.94304 1.93966
C 4.14906 0.28435 0.92566
H 4.82167 0.61302 1.70887
C 4.62411 -0.33135 -0.23128
H 5.68709 -0.49584 -0.37015
C 3.72038 -0.71933 -1.22342
H 4.05962 -1.17701 -2.14556
C 2.36493 -0.50154 -1.01807
C 1.28118 -0.84197 -2.01646
H 1.65837 -0.7976 -3.04366
H 0.88987 -1.84843 -1.84572
C 0.64414 1.49583 -2.21159
H 0.39588 1.67323 -3.2618
H 1.73271 1.54698 -2.12868
C 0.0506 2.52944 -1.30262
C -0.14812 3.85417 -1.67631
H 0.07058 4.16583 -2.69139
C -0.62382 4.75724 -0.72713
H -0.78852 5.79552 -0.99464
C -0.88497 4.30611 0.5666
H -1.25328 4.97541 1.33518
C -0.67887 2.96536 0.86538
H -0.87027 2.54208 1.84322
C -1.11322 -0.17691 -2.44304
H -0.95536 -0.73401 -3.37185
H -1.57534 0.77518 -2.71224
C -2.04329 -0.94303 -1.51315
H -1.69954 -1.97208 -1.41155
C -3.51911 -0.86383 -1.9706
H -3.57063 -0.56555 -3.02105
H -4.00251 -1.83926 -1.88891
C -4.18996 0.1823 -1.03949
H -4.91768 -0.29631 -0.3794
H -4.7173 0.95784 -1.59847
C -3.03282 0.78869 -0.23158
H -2.5886 1.62983 -0.76209
H -3.29831 1.12067 0.77275
C -2.40168 -1.30369 0.88144
H -3.36626 -1.76169 0.64641
H -2.48638 -0.78234 1.83856
C -1.31937 -2.3363 0.95734
C -1.56189 -3.66787 1.27435
H -2.57849 -3.99796 1.45411
C -0.48678 -4.55009 1.36167
H -0.6496 -5.59317 1.60981
C 0.79882 -4.0646 1.13031
H 1.66945 -4.70582 1.19756
C 0.96211 -2.72514 0.7978
H 1.94702 -2.32432 0.60819
C 1.35296 -3.19236 5.57417
H 1.61537 -3.68068 6.52065
H 1.44718 -3.95367 4.78634
C 2.32869 -2.04445 5.28751
H 2.30058 -1.32541 6.11736

H 3.35822 -2.41626 5.21954
C -0.09658 -2.69333 5.62131
H -0.78941 -3.52638 5.79128
H -0.21862 -1.99567 6.46083
C -0.46905 -1.97727 4.3021
H -1.49422 -1.5927 4.34996
H -0.43169 -2.71719 3.49652
C 1.96175 -1.31236 3.97379
H 2.11343 -2.00519 3.1366
H 2.63547 -0.46257 3.82285
C 0.51678 -0.8639 4.02596
H 0.36921 0.00273 4.68132
H 0.17952 -0.29185 2.87274

^{5}**TS**

Fe -0.07756 0.34082 0.51907
O -0.30654 0.7156 2.18221
N 2.0202 0.11079 0.21533
N 0.20159 0.28689 -1.71961
N -0.27983 2.37999 -0.06204
N -2.05912 -0.31512 -0.08981
N -0.08443 -1.87532 0.88148
C 2.94898 0.3825 1.14809
H 2.58967 0.86414 2.04965
C 4.28645 0.05472 0.96477
H 5.01067 0.28326 1.73746
C 4.66202 -0.56809 -0.22646
H 5.69787 -0.83816 -0.40188
C 3.69662 -0.83008 -1.1997
H 3.96193 -1.29653 -2.14165
C 2.37516 -0.47902 -0.94768
C 1.25009 -0.73197 -1.9275
H 1.62535 -0.72288 -2.95756
H 0.81365 -1.71871 -1.74524
C 0.67462 1.62334 -2.15473
H 0.49741 1.77363 -3.22502
H 1.75607 1.67789 -1.99895
C 0.0274 2.7072 -1.33426
C -0.19106 3.99142 -1.82289
H 0.04996 4.2243 -2.85397
C -0.71785 4.95846 -0.9678
H -0.89784 5.96551 -1.32896
C -1.0119 4.61635 0.35305
H -1.4197 5.34107 1.04781
C -0.78152 3.31249 0.77021
H -0.99544 2.97158 1.77573
C -1.09866 -0.0357 -2.35592
H -0.94692 -0.52393 -3.32547
H -1.6049 0.91223 -2.55238
C -1.98689 -0.90838 -1.47244
H -1.56459 -1.9108 -1.39369
C -3.44537 -0.92448 -1.98838
H -3.47551 -0.666 -3.05019
H -3.87784 -1.92197 -1.88844
C -4.20432 0.11003 -1.11288
H -4.97946 -0.381 -0.51942
H -4.69109 0.88286 -1.71132
C -3.12718 0.72613 -0.2016
H -2.68927 1.61433 -0.65753
H -3.47599 0.99797 0.79548
C -2.44799 -1.35138 0.89863
H -3.36636 -1.86698 0.60076
H -2.64166 -0.84114 1.84729
C -1.33117 -2.34071 1.09146
C -1.5687 -3.64607 1.51376
H -2.5875 -3.98833 1.65676
C -0.48338 -4.48492 1.75665
H -0.64219 -5.50344 2.09424
C 0.80718 -3.99494 1.55724
H 1.68167 -4.60983 1.73402
C 0.96196 -2.68694 1.11483
H 1.94721 -2.27464 0.94217
C 0.98268 -2.22836 4.96955
H 1.22872 -3.21615 4.56231
H 1.15338 -2.27898 6.05433
C -0.49269 -1.90385 4.70018
H -0.67676 -1.94546 3.62105
H -1.14275 -2.65697 5.16005
C 1.90527 -1.16475 4.36007
H 2.95506 -1.3908 4.57958
H 1.79967 -1.18645 3.26826
C 1.55594 0.24119 4.86857
H 2.17218 1.00549 4.37955
H 1.78984 0.30505 5.94441
C -0.86884 -0.50597 5.21002
H -0.82218 -0.49388 6.31205
H -1.90204 -0.25598 4.94184
C 0.08201 0.56276 4.70343
H -0.18412 1.57573 5.02392
H -0.10929 0.62643 3.49003

^{3}**IM**

Fe -0.23404 0.53954 -0.36956
O -0.43657 0.92074 1.37625
N 1.74772 0.47606 -0.42662
N 0.10492 0.47316 -2.41128
N -0.4066 2.47972 -0.76529
N -2.15548 0.08155 -0.84654
N -0.25282 -1.45399 0.12451
C 2.56346 0.88382 0.55818
H 2.08095 1.35887 1.40312
C 3.93791 0.68328 0.48845
H 4.57023 1.01679 1.30254
C 4.46985 0.05187 -0.63485
H 5.53788 -0.11824 -0.71597
C 3.61683 -0.34854 -1.6661
H 4.00023 -0.82384 -2.56166
C 2.25356 -0.12393 -1.53318
C 1.21823 -0.49632 -2.56943
H 1.63513 -0.46927 -3.58161
H 0.83103 -1.50328 -2.39235
C 0.55747 1.82576 -2.8606
H 0.35329 1.96199 -3.92593
H 1.64042 1.89554 -2.73043
C -0.09058 2.87931 -2.01409
C -0.3007 4.18682 -2.43719
H -0.05187 4.47143 -3.45323
C -0.82808 5.10845 -1.53352
H -1.00343 6.13434 -1.83933
C -1.12644 4.69304 -0.23589
H -1.53428 5.37912 0.49708
C -0.90696 3.36632 0.11351
H -1.12448 2.96942 1.09656
C -1.16645 0.11381 -3.10774
H -0.95891 -0.47663 -4.00527
H -1.62785 1.04752 -3.4349
C -2.12644 -0.62804 -2.18736
H -1.77549 -1.64692 -2.02636
C -3.58128 -0.58825 -2.71047
H -3.59097 -0.34857 -3.77706
H -4.05944 -1.56287 -2.59517
C -4.29867 0.49977 -1.8662

H -5.05602 0.05087 -1.21892
H -4.80052 1.24393 -2.48773
C -3.18357 1.15249 -1.03486
H -2.72106 1.97206 -1.58323
H -3.49727 1.52857 -0.06055
C -2.58617 -0.87358 0.21197
H -3.53766 -1.35039 -0.03846
H -2.71364 -0.30145 1.1345
C -1.50465 -1.89575 0.39039
C -1.75684 -3.20188 0.79447
H -2.77738 -3.51716 0.97869
C -0.68736 -4.08131 0.95245
H -0.85862 -5.10483 1.26731
C 0.60323 -3.61966 0.70085
H 1.4691 -4.2604 0.81659
C 0.77799 -2.30388 0.29
H 1.76761 -1.91973 0.09008
C 1.35807 -3.21105 4.82363
H 1.60615 -3.89097 5.6479
H 1.28157 -3.82791 3.91639
C 2.47858 -2.17882 4.63992
H 2.61076 -1.61346 5.57202
H 3.43254 -2.67574 4.42494
C 0.00659 -2.53509 5.09016
H -0.78869 -3.28404 5.18951
H 0.05403 -1.98458 6.03909
C -0.35094 -1.54735 3.94796
H -1.28092 -1.01553 4.17584
H -0.53553 -2.14788 3.04657
C 2.13716 -1.19116 3.49247
H 2.15318 -1.76391 2.5535
H 2.90824 -0.41786 3.41118
C 0.78136 -0.59456 3.71269
H 0.72001 0.40085 4.1491
H -0.07183 0.22773 1.96784

^{5}IM

Fe -0.13973 0.47605 0.61711
O -0.47519 1.06034 2.29618
N 1.96253 0.20866 0.37262
N 0.21233 0.28471 -1.63379
N -0.32335 2.48737 -0.14261
N -2.10298 -0.21736 -0.05105
N -0.17229 -1.71494 1.09796
C 2.86349 0.52688 1.31938
H 2.48091 1.06341 2.17968
C 4.20286 0.1786 1.20013
H 4.90286 0.44546 1.98275
C 4.61163 -0.51447 0.05968
H 5.65003 -0.8021 -0.06521
C 3.67632 -0.82446 -0.92863
H 3.9681 -1.34618 -1.83299
C 2.35041 -0.45009 -0.74206
C 1.25629 -0.75302 -1.74219
H 1.66641 -0.81237 -2.75761
H 0.80452 -1.72346 -1.5148
C 0.71126 1.58692 -2.13568
H 0.58209 1.66452 -3.22075
H 1.78508 1.65022 -1.93598
C 0.03135 2.72482 -1.42101
C -0.17313 3.96804 -2.01112
H 0.10891 4.12704 -3.04587
C -0.74037 4.98997 -1.25089
H -0.91165 5.96632 -1.69178
C -1.08628 4.74166 0.07829
H -1.52714 5.51121 0.70099
C -0.86403 3.47328 0.5983
H -1.11413 3.20346 1.6173
C -1.06746 -0.06772 -2.29319
H -0.88752 -0.60861 -3.22948
H -1.56216 0.86997 -2.55736
C -1.98729 -0.88886 -1.39227
H -1.56982 -1.88549 -1.24355
C -3.42766 -0.93554 -1.95402
H -3.42282 -0.74524 -3.03048
H -3.86624 -1.92428 -1.80472
C -4.21058 0.1532 -1.16999
H -5.01027 -0.29766 -0.57719
H -4.66986 0.89114 -1.83093
C -3.16239 0.81813 -0.25855
H -2.70513 1.6777 -0.74847
H -3.54375 1.14748 0.70885
C -2.53017 -1.19161 0.98567
H -3.44887 -1.7109 0.69633
H -2.73656 -0.62622 1.89966
C -1.42906 -2.18164 1.24805
C -1.68478 -3.49112 1.64418
H -2.70889 -3.83379 1.73948
C -0.61109 -4.33839 1.91175
H -0.78546 -5.36235 2.22412
C 0.68721 -3.85029 1.76829
H 1.55288 -4.47141 1.96509
C 0.8623 -2.53449 1.35785
H 1.85423 -2.12244 1.23185
C 1.17353 -2.30544 5.15198
H 1.47869 -3.26701 4.72246
H 1.36374 -2.36505 6.23315
C -0.32375 -2.07602 4.91009
H -0.52071 -2.095 3.83296
H -0.9146 -2.88643 5.35164
C 2.0145 -1.17229 4.55066
H 3.08079 -1.33612 4.7433
H 1.88893 -1.17455 3.461
C 1.59068 0.19967 5.10449
H 2.10927 1.01491 4.58478
H 1.92104 0.26518 6.15884
C -0.7886 -0.72309 5.47542
H -0.78471 -0.78391 6.58081
H -1.82766 -0.51552 5.19253
C 0.10808 0.40448 5.06932
H -0.26697 1.42091 5.18234
H -0.26346 0.62988 3.1647

^{3}PC$_{reb}$

Fe -0.07989 0.35023 -0.3043
O 0.33687 0.28551 1.88408
N 2.00045 0.29077 -0.68749
N 0.12578 0.60761 -2.55361
N -0.31048 2.32749 -0.51039
N -2.07157 -0.11059 -0.85878
N -0.07159 -1.66602 -0.02855
C 2.9664 0.52472 0.21668
H 2.6294 0.8676 1.18737
C 4.31252 0.31635 -0.06513
H 5.06049 0.5168 0.69323
C 4.66535 -0.15103 -1.33064
H 5.70529 -0.3272 -1.58474
C 3.66306 -0.37556 -2.27444
H 3.90181 -0.71882 -3.27519
C 2.33711 -0.14406 -1.92227
C 1.18937 -0.34454 -2.89115
H 1.54245 -0.23154 -3.92539

H 0.78798 -1.35881 -2.7921
C 0.54699 2.00393 -2.76435
H 0.28497 2.36017 -3.7678
H 1.63647 2.06049 -2.68704
C -0.05045 2.89791 -1.70913
C -0.30657 4.24589 -1.94613
H -0.09739 4.66334 -2.9249
C -0.83475 5.03399 -0.92627
H -1.03997 6.08581 -1.09448
C -1.11021 4.44121 0.30516
H -1.53749 5.00487 1.12613
C -0.84026 3.08925 0.46635
H -1.07286 2.58227 1.39134
C -1.19216 0.32959 -3.14756
H -1.09825 -0.09867 -4.1535
H -1.70995 1.2854 -3.26049
C -2.04406 -0.60381 -2.28338
H -1.61052 -1.60525 -2.28864
C -3.51909 -0.60072 -2.7479
H -3.58872 -0.28323 -3.79194
H -3.94548 -1.60442 -2.68917
C -4.24899 0.37853 -1.78869
H -5.01083 -0.14637 -1.20663
H -4.74841 1.18935 -2.32346
C -3.14144 0.93031 -0.87203
H -2.71542 1.84631 -1.28123
H -3.46469 1.13857 0.14903
C -2.44399 -1.22289 0.05335
H -3.36314 -1.72869 -0.26055
H -2.63041 -0.7903 1.04218
C -1.30649 -2.19989 0.13499
C -1.49909 -3.5549 0.38008
H -2.50628 -3.94086 0.48965
C -0.38978 -4.3923 0.47852
H -0.51643 -5.45232 0.67053
C 0.88161 -3.84319 0.32019
H 1.77523 -4.45253 0.38651
C 0.99852 -2.48271 0.06628
H 1.96861 -2.02391 -0.05994
C -0.68928 1.14905 5.9637
H -0.87444 1.72935 6.87503
H -0.87152 0.09458 6.21471
C 0.76982 1.31791 5.51861
H 0.98494 2.38544 5.37133
H 1.454 0.9626 6.29703
C -1.66201 1.57963 4.85723
H -2.69903 1.40917 5.16679
H -1.5581 2.6593 4.67967
C -1.38211 0.82453 3.54865
H -2.05514 1.16349 2.75129
H -1.57022 -0.24916 3.69207
C 1.04625 0.56455 4.2076
H 0.92423 -0.51627 4.37435
H 2.07544 0.72999 3.86958
C 0.0717 1.00589 3.11957
H 0.2682 2.05239 2.87057
H 0.39334 -0.66129 2.09695

^{5}PC$_{reb}$

Fe -0.0834 0.44611 -0.10665
O 0.4351 0.33152 1.99997
N 2.04219 0.135 -0.56808
N 0.10475 0.67258 -2.3506
N -0.33591 2.59596 -0.4318
N -2.14993 -0.11459 -0.73331
N -0.18418 -1.8147 0.16596
C 3.0581 0.21045 0.30887
H 2.799 0.56368 1.30012
C 4.35544 -0.16205 -0.02549
H 5.14727 -0.08894 0.71098
C 4.60325 -0.62505 -1.31799
H 5.60315 -0.92434 -1.61452
C 3.55164 -0.6879 -2.23164
H 3.71204 -1.02712 -3.24916
C 2.27857 -0.30121 -1.82264
C 1.08207 -0.35291 -2.74694
H 1.40156 -0.23382 -3.79094
H 0.60174 -1.33358 -2.66358
C 0.61813 2.03382 -2.60307
H 0.46468 2.33228 -3.64746
H 1.69787 2.0337 -2.42651
C -0.01664 3.035 -1.6677
C -0.23353 4.35612 -2.05438
H 0.02311 4.67086 -3.06001
C -0.78373 5.25108 -1.13932
H -0.96073 6.28374 -1.42128
C -1.11482 4.79555 0.13683
H -1.55532 5.45211 0.87809
C -0.87893 3.46166 0.44373
H -1.14429 3.05937 1.41328
C -1.22892 0.48519 -2.95777
H -1.14359 0.15652 -4.00147
H -1.72101 1.46104 -2.97378
C -2.09767 -0.50594 -2.18222
H -1.66867 -1.50732 -2.25138
C -3.56276 -0.46206 -2.67783
H -3.60973 -0.0872 -3.70398
H -3.99737 -1.46409 -2.68236
C -4.30303 0.46783 -1.67872
H -5.09539 -0.07618 -1.15834
H -4.76656 1.32386 -2.17417
C -3.21418 0.93185 -0.69119
H -2.77351 1.87688 -1.00993
H -3.56263 1.05858 0.33534
C -2.54546 -1.27719 0.1009
H -3.46993 -1.74217 -0.25823
H -2.74105 -0.90117 1.11129
C -1.44124 -2.30106 0.15874
C -1.70537 -3.66681 0.22707
H -2.73049 -4.0199 0.2014
C -0.63789 -4.55802 0.32556
H -0.8189 -5.62641 0.37998
C 0.66217 -4.05441 0.34537
H 1.52312 -4.70884 0.41849
C 0.84277 -2.67809 0.25899
H 1.83583 -2.2448 0.26473
C -0.92514 1.61591 5.86581
H -1.17989 2.28698 6.69417
H -1.14525 0.5937 6.20449
C 0.56962 1.72882 5.53587
H 0.8131 2.77439 5.30115
H 1.17765 1.45067 6.40369
C -1.79142 1.93853 4.64053
H -2.85379 1.80415 4.87169
H -1.65843 2.99488 4.3676
C -1.40811 1.05348 3.44458
H -1.99904 1.3117 2.55837
H -1.62625 0.00169 3.67875
C 0.94804 0.8422 4.33804
H 0.79258 -0.21416 4.60265
H 2.00532 0.96337 4.07756
C 0.07956 1.17783 3.12999

H 0.31605 2.18745 2.78265
H 0.3401 -0.60065 2.26392

^{2}**FP**
Fe -0.23709 0.52627 -0.38371
O -0.42859 0.87865 1.37713
N 1.74537 0.46334 -0.44941
N 0.09787 0.48008 -2.42491
N -0.39508 2.47102 -0.75089
N -2.15437 0.06834 -0.87135
N -0.25862 -1.46028 0.11185
C 2.5608 0.86574 0.53763
H 2.07597 1.32539 1.39008
C 3.93694 0.68058 0.45702
H 4.57111 1.01358 1.26988
C 4.46887 0.06637 -0.67592
H 5.53821 -0.09112 -0.76502
C 3.61424 -0.33429 -1.70567
H 3.99695 -0.79856 -2.60724
C 2.25005 -0.12247 -1.56338
C 1.21025 -0.49 -2.5949
H 1.61969 -0.45721 -3.60975
H 0.82322 -1.49741 -2.41941
C 0.55176 1.83732 -2.86134
H 0.33912 1.9868 -3.92311
H 1.63599 1.90151 -2.74014
C -0.08426 2.88383 -1.99694
C -0.28935 4.19761 -2.40244
H -0.0449 4.49371 -3.41624
C -0.80619 5.11099 -1.48432
H -0.97739 6.14151 -1.77656
C -1.1001 4.68151 -0.19034
H -1.50043 5.36071 0.55306
C -0.88661 3.34927 0.14128
H -1.10458 2.94257 1.12027
C -1.16977 0.12375 -3.12892
H -0.9572 -0.45558 -4.03238
H -1.63545 1.0587 -3.44627
C -2.12427 -0.63237 -2.21617
H -1.76791 -1.65064 -2.06198
C -3.57948 -0.59224 -2.73671
H -3.59043 -0.36013 -3.80489
H -4.06045 -1.56458 -2.61412
C -4.29169 0.50371 -1.89764
H -5.05847 0.06287 -1.25607
H -4.78095 1.25289 -2.5231
C -3.17675 1.14626 -1.05739
H -2.70778 1.96757 -1.59743
H -3.49274 1.51728 -0.08199
C -2.59798 -0.89205 0.17609
H -3.5357 -1.38204 -0.10025
H -2.76489 -0.32121 1.09317
C -1.50858 -1.90036 0.38947
C -1.75174 -3.18675 0.85544
H -2.77036 -3.50217 1.05036
C -0.675 -4.04641 1.06236
H -0.83939 -5.05477 1.42638
C 0.6128 -3.58846 0.79069
H 1.48227 -4.21814 0.93698
C 0.77995 -2.29294 0.31916
H 1.76739 -1.90933 0.10742
H -0.22768 0.09674 1.91901

^{6}**FP**
Fe -0.2299 0.66257 -0.14138
O -0.47013 1.12612 1.62077
N 1.86678 0.4301 -0.40763
N 0.10194 0.61657 -2.39166
N -0.43604 2.70924 -0.74381
N -2.19637 -0.00556 -0.83172
N -0.25266 -1.51806 0.28325
C 2.76729 0.70509 0.55372
H 2.37495 1.1595 1.45632
C 4.1152 0.40982 0.39334
H 4.81755 0.64235 1.18476
C 4.53096 -0.18608 -0.79836
H 5.57633 -0.42978 -0.95453
C 3.59339 -0.45729 -1.79595
H 3.88948 -0.90606 -2.73725
C 2.2601 -0.13715 -1.56962
C 1.16106 -0.39709 -2.5747
H 1.55834 -0.38592 -3.59621
H 0.72637 -1.38671 -2.40218
C 0.57785 1.95752 -2.81004
H 0.43081 2.10657 -3.88491
H 1.65371 2.01956 -2.62212
C -0.10228 3.03654 -2.00882
C -0.32503 4.31616 -2.50653
H -0.06265 4.54985 -3.53217
C -0.88443 5.27849 -1.66645
H -1.06874 6.28211 -2.03482
C -1.20595 4.93654 -0.35203
H -1.63969 5.65807 0.33016
C -0.96983 3.63645 0.07418
H -1.2044 3.29646 1.07576
C -1.17572 0.28346 -3.06635
H -0.99 -0.20195 -4.03119
H -1.68577 1.22614 -3.27687
C -2.07784 -0.60066 -2.2092
H -1.64649 -1.59846 -2.11665
C -3.52017 -0.63278 -2.76647
H -3.52117 -0.38826 -3.83188
H -3.94933 -1.6316 -2.66581
C -4.30899 0.40843 -1.92566
H -5.10427 -0.07785 -1.3555
H -4.77431 1.17652 -2.5467
C -3.26419 1.03315 -0.98282
H -2.81417 1.92057 -1.42795
H -3.64539 1.30876 0.0012
C -2.62038 -1.04188 0.14448
H -3.51893 -1.56753 -0.19242
H -2.86681 -0.52966 1.07982
C -1.5018 -2.01779 0.38698
C -1.73453 -3.34506 0.73236
H -2.75159 -3.71618 0.78816
C -0.64635 -4.17381 0.99926
H -0.80266 -5.21256 1.26991
C 0.64298 -3.64993 0.90839
H 1.51782 -4.25743 1.10756
C 0.79735 -2.31833 0.5459
H 1.78029 -1.87464 0.46406
H -0.42505 0.40829 2.27443

CYH + **2**
^{3}**RC**
Fe 0.08947 0.00742 0.12174
O 0.11573 -0.09493 1.74505
N 2.09441 0.05191 0.03488
N 0.20054 -0.18788 -2.02923
N 0.3211 1.98289 0.13019
N -0.15762 -1.96622 -0.09189
C 0.96149 1.2819 -5.01705

H 1.815 1.70362 -4.49234
C 1.00734 1.13979 -6.40451
H 1.89942 1.43623 -6.94772
C -0.09535 0.62695 -7.09051
H -0.06217 0.51784 -8.17031
C -1.24722 0.26907 -6.38589
H -2.11426 -0.11355 -6.91571
C -1.29046 0.41157 -4.99905
H -2.19682 0.14858 -4.46072
C -0.18165 0.90515 -4.29793
C -0.21677 1.04164 -2.79487
H 0.44062 1.85088 -2.4783
H -1.22697 1.28019 -2.457
C 1.62259 -0.55704 -2.28795
H 1.87188 -0.41129 -3.34194
H 1.73884 -1.62206 -2.07824
C 2.55419 0.24044 -1.39726
H 2.48675 1.30378 -1.63176
C 4.01226 -0.27893 -1.44755
H 4.12894 -0.96619 -2.28986
H 4.71536 0.54177 -1.5985
C 4.25007 -0.99695 -0.09458
H 4.7332 -1.96804 -0.21857
H 4.88467 -0.39604 0.56182
C 2.84832 -1.15883 0.50056
H 2.35577 -2.0395 0.09175
H 2.80738 -1.21423 1.58809
C 2.48339 1.24184 0.85136
H 3.52338 1.52181 0.66733
H 2.37762 0.9681 1.90433
C 1.55086 2.36899 0.53405
C 1.88221 3.71198 0.6683
H 2.88076 3.98995 0.9848
C 0.91434 4.67609 0.39232
H 1.14822 5.73018 0.49295
C -0.35514 4.26736 -0.01648
H -1.13664 4.98271 -0.24183
C -0.61731 2.9097 -0.13498
H -1.586 2.54768 -0.44777
C -0.76394 -3.75255 -1.55494
H -1.03438 -4.07931 -2.55236
C -0.65894 -4.65663 -0.49986
H -0.84825 -5.71192 -0.664
C -0.30658 -4.18754 0.76609
H -0.21704 -4.85618 1.61387
C -0.06271 -2.83175 0.93429
H 0.2129 -2.40124 1.88852
C -0.51777 -2.40485 -1.3166
C -0.67067 -1.34247 -2.36684
H -0.44346 -1.73393 -3.36265
H -1.70741 -0.99568 -2.37926
C -3.0354 0.15398 0.1086
C -4.48247 0.18006 0.20896
H -4.85602 -0.83719 0.35592
H -4.77452 0.80323 1.05888
H -4.90527 0.5957 -0.71017
N -1.88424 0.14155 0.02382
C -0.54816 2.65303 3.71231
H 0.21014 2.10981 4.29024
H -0.15599 2.74058 2.69173
C -0.75679 4.05909 4.29149
H -1.03952 3.97443 5.35104
H 0.18286 4.62522 4.2598
C -1.858 1.85372 3.67537
H -1.68314 0.8787 3.20681
H -2.19403 1.66378 4.70538
C -2.95865 2.61666 2.92557
H -3.89899 2.05054 2.95419
H -2.6749 2.7101 1.86727
C -1.85862 4.81766 3.53758
H -1.52742 4.99959 2.5055
H -2.0223 5.80232 3.99312
C -3.17061 4.02042 3.51018
H -3.55612 3.92591 4.53585
H -3.9328 4.56119 2.93518

[5]RC

Fe 0.03345 -0.01231 0.1462
O 0.02404 -0.13154 1.76154
N 2.20508 0.01621 0.03988
N 0.2341 -0.20441 -2.01938
N 0.42859 2.07674 0.15209
N -0.42561 -1.9969 -0.13355
C 1.24348 1.26291 -4.92363
H 2.1056 1.59382 -4.35038
C 1.34012 1.14985 -6.31095
H 2.27858 1.37838 -6.8067
C 0.22899 0.75277 -7.05767
H 0.30177 0.66627 -8.13757
C -0.98056 0.48128 -6.41377
H -1.85232 0.18858 -6.99097
C -1.07462 0.59461 -5.02672
H -2.02361 0.3991 -4.53528
C 0.03989 0.97308 -4.26583
C -0.04948 1.07827 -2.76315
H 0.65853 1.8197 -2.39577
H -1.04855 1.39056 -2.4541
C 1.63096 -0.69044 -2.23756
H 1.88507 -0.63038 -3.29931
H 1.66073 -1.74648 -1.96093
C 2.63806 0.08279 -1.40053
H 2.65664 1.13183 -1.7
C 4.04801 -0.5631 -1.45375
H 4.08302 -1.3149 -2.24715
H 4.81143 0.18319 -1.67946
C 4.26586 -1.20181 -0.05873
H 4.67941 -2.21042 -0.12145
H 4.95292 -0.59927 0.54185
C 2.87001 -1.21161 0.57469
H 2.29532 -2.07871 0.24227
H 2.85365 -1.19113 1.66442
C 2.61258 1.22973 0.78655
H 3.65594 1.49079 0.58575
H 2.51716 1.00843 1.85377
C 1.70826 2.38516 0.44602
C 2.14345 3.70626 0.47309
H 3.18195 3.92358 0.69577
C 1.22905 4.72647 0.21694
H 1.54733 5.76308 0.23604
C -0.09654 4.39649 -0.06387
H -0.84084 5.15731 -0.26564
C -0.45988 3.05662 -0.08866
H -1.47338 2.74793 -0.30748
C -1.06025 -3.68714 -1.69212
H -1.28781 -3.96878 -2.71377
C -1.09194 -4.62292 -0.65835
H -1.34783 -5.65595 -0.86792
C -0.78752 -4.22098 0.64336
H -0.80144 -4.92087 1.47015
C -0.45638 -2.89323 0.87093
H -0.20848 -2.50975 1.85331
C -0.7283 -2.37057 -1.39729

C -0.72559 -1.26986 -2.42002
H -0.48903 -1.65493 -3.41577
H -1.72327 -0.82437 -2.46504
C -3.28205 0.45253 -0.05586
C -4.72574 0.51171 0.07896
H -5.15722 1.00462 -0.79682
H -5.12669 -0.50258 0.16049
H -4.98219 1.07763 0.97926
N -2.1307 0.41537 -0.16269
C -0.18153 3.77952 3.62406
H 0.20015 4.79987 3.75508
H 0.38107 3.34004 2.78942
C -1.67364 3.80901 3.26541
H -2.22461 4.32895 4.06271
H -1.83275 4.38587 2.34602
C 0.07116 2.9434 4.88618
H 1.14517 2.90728 5.10911
H -0.41367 3.43131 5.74467
C -0.48772 1.52203 4.72899
H -0.33024 0.94679 5.65041
H 0.0627 1.00611 3.93014
C -2.23468 2.38897 3.1086
H -1.74811 1.90204 2.25416
H -3.30914 2.42713 2.88597
C -1.97931 1.54289 4.36406
H -2.55236 1.96179 5.20448
H -2.34574 0.51975 4.21045

^{3}TS

Fe 0.07541 0.10474 -0.05784
O 0.10715 0.02833 1.69075
N 2.09533 0.11485 -0.10478
N 0.2044 -0.16564 -2.16715
N 0.32986 2.0817 -0.06512
N -0.15946 -1.87838 -0.17488
C 0.94534 1.1844 -5.21641
H 1.81216 1.61009 -4.71771
C 0.97035 0.99448 -6.59874
H 1.85954 1.25815 -7.16326
C -0.14912 0.4762 -7.2527
H -0.13217 0.32995 -8.32851
C -1.29673 0.16091 -6.5213
H -2.1767 -0.22574 -7.02639
C -1.31883 0.35146 -5.13973
H -2.22171 0.12139 -4.58078
C -0.19341 0.85078 -4.46971
C -0.2088 1.0385 -2.97156
H 0.46034 1.85139 -2.68919
H -1.21233 1.29957 -2.63079
C 1.6268 -0.54802 -2.41132
H 1.8792 -0.4307 -3.46814
H 1.735 -1.6081 -2.17432
C 2.5628 0.26291 -1.53632
H 2.50628 1.32024 -1.80005
C 4.01747 -0.26917 -1.57223
H 4.12953 -0.9836 -2.39262
H 4.72737 0.54084 -1.74873
C 4.24861 -0.94633 -0.19736
H 4.73015 -1.92198 -0.28968
H 4.88291 -0.3269 0.44236
C 2.84255 -1.08257 0.39564
H 2.3483 -1.97292 0.00985
H 2.79619 -1.10865 1.48425
C 2.48497 1.32444 0.67716
H 3.53103 1.59023 0.50493
H 2.35339 1.0835 1.73486
C 1.57249 2.45528 0.31291
C 1.94047 3.79448 0.38463
H 2.95019 4.05897 0.67684
C 0.99394 4.77222 0.08458
H 1.25612 5.82317 0.13918
C -0.29222 4.37967 -0.28757
H -1.06008 5.10541 -0.52691
C -0.58485 3.02453 -0.35749
H -1.56341 2.67494 -0.65496
C -0.74531 -3.71898 -1.58095
H -1.00827 -4.08185 -2.56802
C -0.63913 -4.58603 -0.49481
H -0.81912 -5.64791 -0.62392
C -0.29791 -4.07108 0.75629
H -0.20812 -4.71097 1.62616
C -0.06477 -2.70764 0.87945
H 0.19948 -2.23601 1.81758
C -0.50996 -2.3625 -1.38477
C -0.66532 -1.33285 -2.46861
H -0.43672 -1.75273 -3.45248
H -1.70246 -0.98823 -2.49219
C -3.03876 0.27578 -0.13791
C -4.48945 0.30158 -0.10566
H -4.84961 -0.39488 0.65703
H -4.83102 1.31254 0.13435
H -4.88341 0.00653 -1.08237
N -1.88443 0.2687 -0.16108
C -0.15116 2.80911 3.40237
H 0.80707 2.44339 3.78796
H 0.04806 3.31201 2.44953
C -0.76562 3.84626 4.37077
H -0.87723 3.39249 5.36477
H -0.07888 4.69443 4.47933
C -1.12035 1.66619 3.18877
H -0.55043 0.85123 2.31425
H -1.17735 0.98978 4.04971
C -2.48322 2.09308 2.68937
H -3.15295 1.2315 2.59375
H -2.39242 2.55866 1.70002
C -2.13518 4.31696 3.86501
H -2.00482 4.84776 2.91071
H -2.57159 5.03447 4.5705
C -3.09085 3.13494 3.6591
H -3.29089 2.65278 4.62541
H -4.05521 3.47786 3.26515

^{5}TS

Fe 0.09991 -0.12358 0.33354
O 0.20337 -0.36877 2.03177
N 2.24128 -0.09202 0.10483
N 0.21861 -0.24439 -1.93637
N 0.52873 2.00086 0.3653
N -0.34501 -2.1512 -0.09251
C 1.10378 1.39018 -4.79351
H 1.97079 1.72371 -4.22902
C 1.16615 1.34111 -6.18686
H 2.08302 1.62225 -6.69613
C 0.04893 0.93969 -6.92192
H 0.09473 0.90287 -8.00622
C -1.1324 0.59895 -6.25875
H -2.00966 0.30139 -6.82534
C -1.19124 0.6483 -4.86583
H -2.11883 0.39706 -4.35883
C -0.07044 1.03178 -4.11655
C -0.11943 1.05641 -2.60695
H 0.574 1.80412 -2.22223

H -1.11851 1.32673 -2.25743
C 1.61396 -0.68046 -2.20374
H 1.86088 -0.55952 -3.26326
H 1.67763 -1.74885 -1.9843
C 2.62058 0.07186 -1.34295
H 2.59663 1.13682 -1.57939
C 4.04682 -0.52195 -1.47823
H 4.09225 -1.18723 -2.34497
H 4.78473 0.26676 -1.63655
C 4.3067 -1.28956 -0.15663
H 4.70622 -2.29144 -0.32728
H 5.02319 -0.75161 0.47014
C 2.93464 -1.34906 0.52627
H 2.35004 -2.19513 0.1601
H 2.96054 -1.40276 1.61452
C 2.69986 1.0657 0.91198
H 3.74141 1.31773 0.69018
H 2.63821 0.77464 1.9647
C 1.81089 2.26155 0.68924
C 2.26485 3.56546 0.86762
H 3.30528 3.74422 1.11499
C 1.36252 4.61889 0.73431
H 1.69248 5.64234 0.87676
C 0.03398 4.34043 0.41042
H -0.699 5.12986 0.2941
C -0.34195 3.01631 0.2276
H -1.35716 2.74803 -0.03653
C -0.9969 -3.76312 -1.72715
H -1.24718 -3.98799 -2.75788
C -0.98647 -4.75903 -0.75172
H -1.23153 -5.78253 -1.01526
C -0.653 -4.42707 0.56273
H -0.63293 -5.17409 1.34744
C -0.3356 -3.10801 0.85537
H -0.06518 -2.77648 1.8505
C -0.67719 -2.45908 -1.3627
C -0.72733 -1.31006 -2.33587
H -0.53342 -1.66061 -3.35482
H -1.73579 -0.88483 -2.32419
C -3.16378 0.37759 0.2404
C -4.60108 0.46689 0.41552
H -5.03233 1.07746 -0.38276
H -5.03586 -0.53622 0.38289
H -4.8183 0.92641 1.38425
N -2.01606 0.31563 0.10966
C 0.35973 2.49376 4.16138
H 1.03982 3.20343 4.64634
H 0.75763 2.31153 3.15615
C -1.04852 3.09253 4.04359
H -1.4123 3.36613 5.04427
H -1.014 4.01647 3.45395
C 0.34717 1.16831 4.93578
H 1.34508 0.71481 4.95247
H 0.07589 1.36325 5.98687
C -0.66931 0.18899 4.37566
H -0.67133 -0.78013 4.88635
H -0.26095 -0.10361 3.26835
C -2.02487 2.0976 3.40206
H -1.71166 1.90732 2.36935
H -3.03363 2.52448 3.35381
C -2.05899 0.76313 4.16049
H -2.50855 0.92125 5.15502
H -2.6977 0.03588 3.64523

^{3}IM

Fe 0.11762 -0.00411 -0.06192
O 0.18434 -0.11678 1.7288
N 2.13351 0.04476 -0.12575
N 0.2087 -0.19972 -2.16181
N 0.32748 1.97456 -0.00881
N -0.0765 -1.9827 -0.23034
C 0.90046 1.29449 -5.16036
H 1.75022 1.73617 -4.6462
C 0.93036 1.15629 -6.54864
H 1.80715 1.47565 -7.10366
C -0.16886 0.61749 -7.22016
H -0.14821 0.51093 -8.30055
C -1.30152 0.23027 -6.50008
H -2.16631 -0.17261 -7.01845
C -1.32872 0.36933 -5.11245
H -2.22075 0.08339 -4.56216
C -0.2224 0.88818 -4.42576
C -0.2384 1.02239 -2.92223
H 0.41225 1.84054 -2.61423
H -1.24629 1.24622 -2.56838
C 1.63427 -0.55022 -2.44388
H 1.86335 -0.39308 -3.50067
H 1.76273 -1.61565 -2.2449
C 2.57481 0.24555 -1.55915
H 2.50026 1.30988 -1.78845
C 4.03519 -0.26676 -1.63929
H 4.13487 -0.96883 -2.47194
H 4.73052 0.5542 -1.82239
C 4.30785 -0.9616 -0.2815
H 4.81663 -1.92057 -0.39922
H 4.93317 -0.33512 0.36025
C 2.91505 -1.14923 0.32799
H 2.43718 -2.0396 -0.07841
H 2.88539 -1.20937 1.41573
C 2.50591 1.23762 0.69032
H 3.54351 1.53345 0.51531
H 2.38932 0.9595 1.74041
C 1.56465 2.36009 0.37642
C 1.90136 3.70411 0.49865
H 2.90612 3.98035 0.79685
C 0.93199 4.67064 0.238
H 1.17085 5.7244 0.33092
C -0.34689 4.26392 -0.14481
H -1.13108 4.98096 -0.35559
C -0.61065 2.90642 -0.26099
H -1.58312 2.54504 -0.56432
C -0.67166 -3.78832 -1.67584
H -0.95563 -4.12646 -2.66578
C -0.52313 -4.68482 -0.61868
H -0.69074 -5.74511 -0.77468
C -0.1563 -4.20207 0.638
H -0.03394 -4.86627 1.48536
C 0.05908 -2.83932 0.79702
H 0.33793 -2.38872 1.74095
C -0.45133 -2.4351 -1.44498
C -0.64759 -1.37212 -2.48886
H -0.42919 -1.75109 -3.49132
H -1.69085 -1.0451 -2.48038
C -2.99738 0.11244 -0.01008
C -4.44697 0.10245 0.06315
H -4.78925 -0.8851 0.38528
H -4.78165 0.8556 0.78191
H -4.86429 0.32934 -0.92205
N -1.84447 0.12724 -0.07599
C -0.31085 2.73026 3.56141
H 0.52467 2.26897 4.09824
H 0.06164 3.01211 2.56718

C -0.76208 4.03534 4.26889
H -1.00653 3.81077 5.31554
H 0.06761 4.75278 4.27682
C -1.4664 1.78566 3.42992
H -0.48746 0.46724 2.14828
H -1.56361 0.98029 4.15612
C -2.71128 2.32559 2.79742
H -3.52164 1.58898 2.82184
H -2.52346 2.57611 1.74247
C -1.99116 4.63646 3.57447
H -1.71714 4.94127 2.55415
H -2.31163 5.54458 4.09959
C -3.1504 3.63314 3.50943
H -3.48191 3.39116 4.52782
H -4.00992 4.06635 2.98352

^{5}IM

Fe 0.16315 -0.24569 0.444
O 0.32479 -0.61472 2.20779
N 2.30908 -0.18987 0.12999
N 0.23402 -0.27373 -1.86616
N 0.59035 1.88001 0.50847
N -0.27157 -2.26882 -0.09958
C 1.02838 1.4675 -4.68348
H 1.91622 1.77127 -4.13479
C 1.04453 1.47664 -6.07899
H 1.94646 1.77264 -6.60622
C -0.09955 1.11442 -6.7928
H -0.08976 1.12307 -7.87862
C -1.26118 0.75425 -6.10558
H -2.15909 0.487 -6.65469
C -1.27364 0.74519 -4.71061
H -2.18613 0.4792 -4.18407
C -0.12582 1.08905 -3.98338
C -0.12574 1.05121 -2.47294
H 0.57947 1.78434 -2.08168
H -1.11363 1.30668 -2.08212
C 1.62437 -0.68923 -2.18202
H 1.84679 -0.52556 -3.24145
H 1.69923 -1.76507 -2.00668
C 2.65011 0.03333 -1.31673
H 2.616 1.10669 -1.51068
C 4.07456 -0.54787 -1.51489
H 4.09793 -1.18117 -2.40625
H 4.80504 0.24938 -1.66508
C 4.37476 -1.36166 -0.23004
H 4.77707 -2.3533 -0.44779
H 5.10342 -0.8413 0.39778
C 3.02106 -1.45622 0.48559
H 2.43277 -2.29269 0.10327
H 3.07635 -1.5511 1.56997
C 2.77696 0.9384 0.97345
H 3.8152 1.20242 0.74948
H 2.72664 0.60868 2.01535
C 1.88203 2.13749 0.8012
C 2.33986 3.43965 0.98131
H 3.38614 3.61623 1.20398
C 1.43587 4.49517 0.877
H 1.77019 5.51705 1.02033
C 0.1007 4.22103 0.57828
H -0.63342 5.01205 0.48211
C -0.28058 2.89875 0.3947
H -1.30107 2.63496 0.14792
C -0.94111 -3.80965 -1.79482
H -1.21264 -3.98817 -2.82924
C -0.89286 -4.85175 -0.87
H -1.13 -5.86479 -1.17765
C -0.53119 -4.57918 0.4506
H -0.48103 -5.36346 1.19672
C -0.22539 -3.27073 0.79953
H 0.06424 -2.98271 1.8031
C -0.62996 -2.5205 -1.3739
C -0.71437 -1.32646 -2.28937
H -0.54642 -1.62766 -3.32869
H -1.7252 -0.91059 -2.23158
C -3.10112 0.25066 0.35251
C -4.54572 0.33797 0.44932
H -4.94231 0.84241 -0.43647
H -4.96807 -0.66878 0.51564
H -4.81556 0.90613 1.3444
N -1.9482 0.19169 0.27771
C 0.19639 2.69266 4.18013
H 0.89077 3.41265 4.62728
H 0.57528 2.47114 3.17598
C -1.20944 3.29535 4.06553
H -1.56531 3.58608 5.06424
H -1.17616 4.20935 3.46091
C 0.18813 1.39608 5.0084
H 1.16902 0.90609 4.98307
H 0.01157 1.65803 6.06925
C -0.88868 0.44605 4.58444
H -0.82423 -0.58327 4.93632
H -0.15464 -0.15647 2.95266
C -2.19094 2.29299 3.44572
H -1.87259 2.07214 2.42051
H -3.19592 2.725 3.37781
C -2.24529 0.97939 4.24506
H -2.78084 1.16943 5.19498
H -2.83855 0.22148 3.71891

^{3}TS

Fe 0.07541 0.10474 -0.05784
O 0.10715 0.02833 1.69075
N 2.09533 0.11485 -0.10478
N 0.2044 -0.16564 -2.16715
N 0.32986 2.0817 -0.06512
N -0.15946 -1.87838 -0.17488
C 0.94534 1.1844 -5.21641
H 1.81216 1.61009 -4.71771
C 0.97035 0.99448 -6.59874
H 1.85954 1.25815 -7.16326
C -0.14912 0.4762 -7.2527
H -0.13217 0.32995 -8.32851
C -1.29673 0.16091 -6.5213
H -2.1767 -0.22574 -7.02639
C -1.31883 0.35146 -5.13973
H -2.22171 0.12139 -4.58078
C -0.19341 0.85078 -4.46971
C -0.2088 1.0385 -2.97156
H 0.46034 1.85139 -2.68919
H -1.21233 1.29957 -2.63079
C 1.6268 -0.54802 -2.41132
H 1.8792 -0.4307 -3.46814
H 1.735 -1.6081 -2.17432
C 2.5628 0.26291 -1.53632
H 2.50628 1.32024 -1.80005
C 4.01747 -0.26917 -1.57223
H 4.12953 -0.9836 -2.39262
H 4.72737 0.54084 -1.74873
C 4.24861 -0.94633 -0.19736
H 4.73015 -1.92198 -0.28968
H 4.88291 -0.3269 0.44236

C 2.84255 -1.08257 0.39564
H 2.3483 -1.97292 0.00985
H 2.79619 -1.10865 1.48425
C 2.48497 1.32444 0.67716
H 3.53103 1.59023 0.50493
H 2.35339 1.0835 1.73486
C 1.57249 2.45528 0.31291
C 1.94047 3.79448 0.38463
H 2.95019 4.05897 0.67684
C 0.99394 4.77222 0.08458
H 1.25612 5.82317 0.13918
C -0.29222 4.37967 -0.28757
H -1.06008 5.10541 -0.52691
C -0.58485 3.02453 -0.35749
H -1.56341 2.67494 -0.65496
C -0.74531 -3.71898 -1.58095
H -1.00827 -4.08185 -2.56802
C -0.63913 -4.58603 -0.49481
H -0.81912 -5.64791 -0.62392
C -0.29791 -4.07108 0.75629
H -0.20812 -4.71097 1.62616
C -0.06477 -2.70764 0.87945
H 0.19948 -2.23601 1.81758
C -0.50996 -2.3625 -1.38477
C -0.66532 -1.33285 -2.46861
H -0.43672 -1.75273 -3.45248
H -1.70246 -0.98823 -2.49219
C -3.03876 0.27578 -0.13791
C -4.48945 0.30158 -0.10566
H -4.84961 -0.39488 0.65703
H -4.83102 1.31254 0.13435
H -4.88341 0.00653 -1.08237
N -1.88443 0.2687 -0.16108
C -0.15116 2.80911 3.40237
H 0.80707 2.44339 3.78796
H 0.04806 3.31201 2.44953
C -0.76562 3.84626 4.37077
H -0.87723 3.39249 5.36477
H -0.07888 4.69443 4.47933
C -1.12035 1.66619 3.18877
H -0.55043 0.85123 2.31425
H -1.17735 0.98978 4.04971
C -2.48322 2.09308 2.68937
H -3.15295 1.2315 2.59375
H -2.39242 2.55866 1.70002
C -2.13518 4.31696 3.86501
H -2.00482 4.84776 2.91071
H -2.57159 5.03447 4.5705
C -3.09085 3.13494 3.6591
H -3.29089 2.65278 4.62541
H -4.05521 3.47786 3.26515

[5]TS

Fe 0.09991 -0.12358 0.33354
O 0.20337 -0.36877 2.03177
N 2.24128 -0.09202 0.10483
N 0.21861 -0.24439 -1.93637
N 0.52873 2.00086 0.3653
N -0.34501 -2.1512 -0.09251
C 1.10378 1.39018 -4.79351
H 1.97079 1.72371 -4.22902
C 1.16615 1.34111 -6.18686
H 2.08302 1.62225 -6.69613
C 0.04893 0.93969 -6.92192
H 0.09473 0.90287 -8.00622
C -1.1324 0.59895 -6.25875
H -2.00966 0.30139 -6.82534
C -1.19124 0.6483 -4.86583
H -2.11883 0.39706 -4.35883
C -0.07044 1.03178 -4.11655
C -0.11943 1.05641 -2.60695
H 0.574 1.80412 -2.22223
H -1.11851 1.32673 -2.25743
C 1.61396 -0.68046 -2.20374
H 1.86088 -0.55952 -3.26326
H 1.67763 -1.74885 -1.9843
C 2.62058 0.07186 -1.34295
H 2.59663 1.13682 -1.57939
C 4.04682 -0.52195 -1.47823
H 4.09225 -1.18723 -2.34497
H 4.78473 0.26676 -1.63655
C 4.3067 -1.28956 -0.15663
H 4.70622 -2.29144 -0.32728
H 5.02319 -0.75161 0.47014
C 2.93464 -1.34906 0.52627
H 2.35004 -2.19513 0.1601
H 2.96054 -1.40276 1.61452
C 2.69986 1.0657 0.91198
H 3.74141 1.31773 0.69018
H 2.63821 0.77464 1.9647
C 1.81089 2.26155 0.68924
C 2.26485 3.56546 0.86762
H 3.30528 3.74422 1.11499
C 1.36252 4.61889 0.73431
H 1.69248 5.64234 0.87676
C 0.03398 4.34043 0.41042
H -0.699 5.12986 0.2941
C -0.34195 3.01631 0.2276
H -1.35716 2.74803 -0.03653
C -0.9969 -3.76312 -1.72715
H -1.24718 -3.98799 -2.75788
C -0.98647 -4.75903 -0.75172
H -1.23153 -5.78253 -1.01526
C -0.653 -4.42707 0.56273
H -0.63293 -5.17409 1.34744
C -0.3356 -3.10801 0.85537
H -0.06518 -2.77648 1.8505
C -0.67719 -2.45908 -1.3627
C -0.72733 -1.31006 -2.33587
H -0.53342 -1.66061 -3.35482
H -1.73579 -0.88483 -2.32419
C -3.16378 0.37759 0.2404
C -4.60108 0.46689 0.41552
H -5.03233 1.07746 -0.38276
H -5.03586 -0.53622 0.38289
H -4.8183 0.92641 1.38425
N -2.01606 0.31563 0.10966
C 0.35973 2.49376 4.16138
H 1.03982 3.20343 4.64634
H 0.75763 2.31153 3.15615
C -1.04852 3.09253 4.04359
H -1.4123 3.36613 5.04427
H -1.014 4.01647 3.45395
C 0.34717 1.16831 4.93578
H 1.34508 0.71481 4.95247
H 0.07589 1.36325 5.98687
C -0.66931 0.18899 4.37566
H -0.67133 -0.78013 4.88635
H -0.26095 -0.10361 3.26835
C -2.02487 2.0976 3.40206
H -1.71166 1.90732 2.36935
H -3.03363 2.52448 3.35381

C -2.05899 0.76313 4.16049
H -2.50855 0.92125 5.15502
H -2.6977 0.03588 3.64523

^{3}IM

Fe 0.11762 -0.00411 -0.06192
O 0.18434 -0.11678 1.7288
N 2.13351 0.04476 -0.12575
N 0.2087 -0.19972 -2.16181
N 0.32748 1.97456 -0.00881
N -0.0765 -1.9827 -0.23034
C 0.90046 1.29449 -5.16036
H 1.75022 1.73617 -4.6462
C 0.93036 1.15629 -6.54864
H 1.80715 1.47565 -7.10366
C -0.16886 0.61749 -7.22016
H -0.14821 0.51093 -8.30055
C -1.30152 0.23027 -6.50008
H -2.16631 -0.17261 -7.01845
C -1.32872 0.36933 -5.11245
H -2.22075 0.08339 -4.56216
C -0.2224 0.88818 -4.42576
C -0.2384 1.02239 -2.92223
H 0.41225 1.84054 -2.61423
H -1.24629 1.24622 -2.56838
C 1.63427 -0.55022 -2.44388
H 1.86335 -0.39308 -3.50067
H 1.76273 -1.61565 -2.2449
C 2.57481 0.24555 -1.55915
H 2.50026 1.30988 -1.78845
C 4.03519 -0.26676 -1.63929
H 4.13487 -0.96883 -2.47194
H 4.73052 0.5542 -1.82239
C 4.30785 -0.9616 -0.2815
H 4.81663 -1.92057 -0.39922
H 4.93317 -0.33512 0.36025
C 2.91505 -1.14923 0.32799
H 2.43718 -2.0396 -0.07841
H 2.88539 -1.20937 1.41573
C 2.50591 1.23762 0.69032
H 3.54351 1.53345 0.51531
H 2.38932 0.9595 1.74041
C 1.56465 2.36009 0.37642
C 1.90136 3.70411 0.49865
H 2.90612 3.98035 0.79685
C 0.93199 4.67064 0.238
H 1.17085 5.7244 0.33092
C -0.34689 4.26392 -0.14481
H -1.13108 4.98096 -0.35559
C -0.61065 2.90642 -0.26099
H -1.58312 2.54504 -0.56432
C -0.67166 -3.78832 -1.67584
H -0.95563 -4.12646 -2.66578
C -0.52313 -4.68482 -0.61868
H -0.69074 -5.74511 -0.77468
C -0.1563 -4.20207 0.638
H -0.03394 -4.86627 1.48536
C 0.05908 -2.83932 0.79702
H 0.33793 -2.38872 1.74095
C -0.45133 -2.4351 -1.44498
C -0.64759 -1.37212 -2.48886
H -0.42919 -1.75109 -3.49132
H -1.69085 -1.0451 -2.48038
C -2.99738 0.11244 -0.01008
C -4.44697 0.10245 0.06315
H -4.78925 -0.8851 0.38528
H -4.78165 0.8556 0.78191
H -4.86429 0.32934 -0.92205
N -1.84447 0.12724 -0.07599
C -0.31085 2.73026 3.56141
H 0.52467 2.26897 4.09824
H 0.06164 3.01211 2.56718
C -0.76208 4.03534 4.26889
H -1.00653 3.81077 5.31554
H 0.06761 4.75278 4.27682
C -1.4664 1.78566 3.42992
H -0.48746 0.46724 2.14828
H -1.56361 0.98029 4.15612
C -2.71128 2.32559 2.79742
H -3.52164 1.58898 2.82184
H -2.52346 2.57611 1.74247
C -1.99116 4.63646 3.57447
H -1.71714 4.94127 2.55415
H -2.31163 5.54458 4.09959
C -3.1504 3.63314 3.50943
H -3.48191 3.39116 4.52782
H -4.00992 4.06635 2.98352

^{5}IM

Fe 0.16315 -0.24569 0.444
O 0.32479 -0.61472 2.20779
N 2.30908 -0.18987 0.12999
N 0.23402 -0.27373 -1.86616
N 0.59035 1.88001 0.50847
N -0.27157 -2.26882 -0.09958
C 1.02838 1.4675 -4.68348
H 1.91622 1.77127 -4.13479
C 1.04453 1.47664 -6.07899
H 1.94646 1.77264 -6.60622
C -0.09955 1.11442 -6.7928
H -0.08976 1.12307 -7.87862
C -1.26118 0.75425 -6.10558
H -2.15909 0.487 -6.65469
C -1.27364 0.74519 -4.71061
H -2.18613 0.4792 -4.18407
C -0.12582 1.08905 -3.98338
C -0.12574 1.05121 -2.47294
H 0.57947 1.78434 -2.08168
H -1.11363 1.30668 -2.08212
C 1.62437 -0.68923 -2.18202
H 1.84679 -0.52556 -3.24145
H 1.69923 -1.76507 -2.00668
C 2.65011 0.03333 -1.31673
H 2.616 1.10669 -1.51068
C 4.07456 -0.54787 -1.51489
H 4.09793 -1.18117 -2.40625
H 4.80504 0.24938 -1.66508
C 4.37476 -1.36166 -0.23004
H 4.77707 -2.3533 -0.44779
H 5.10342 -0.8413 0.39778
C 3.02106 -1.45622 0.48559
H 2.43277 -2.29269 0.10327
H 3.07635 -1.5511 1.56997
C 2.77696 0.9384 0.97345
H 3.8152 1.20242 0.74948
H 2.72664 0.60868 2.01535
C 1.88203 2.13749 0.8012
C 2.33986 3.43965 0.98131
H 3.38614 3.61623 1.20398
C 1.43587 4.49517 0.877
H 1.77019 5.51705 1.02033
C 0.1007 4.22103 0.57828

H -0.63342 5.01205 0.48211
C -0.28058 2.89875 0.3947
H -1.30107 2.63496 0.14792
C -0.94111 -3.80965 -1.79482
H -1.21264 -3.98817 -2.82924
C -0.89286 -4.85175 -0.87
H -1.13 -5.86479 -1.17765
C -0.53119 -4.57918 0.4506
H -0.48103 -5.36346 1.19672
C -0.22539 -3.27073 0.79953
H 0.06424 -2.98271 1.8031
C -0.62996 -2.5205 -1.3739
C -0.71437 -1.32646 -2.28937
H -0.54642 -1.62766 -3.32869
H -1.7252 -0.91059 -2.23158
C -3.10112 0.25066 0.35251
C -4.54572 0.33797 0.44932
H -4.94231 0.84241 -0.43647
H -4.96807 -0.66878 0.51564
H -4.81556 0.90613 1.3444
N -1.9482 0.19169 0.27771
C 0.19639 2.69266 4.18013
H 0.89077 3.41265 4.62728
H 0.57528 2.47114 3.17598
C -1.20944 3.29535 4.06553
H -1.56531 3.58608 5.06424
H -1.17616 4.20935 3.46091
C 0.18813 1.39608 5.0084
H 1.16902 0.90609 4.98307
H 0.01157 1.65803 6.06925
C -0.88868 0.44605 4.58444
H -0.82423 -0.58327 4.93632
H -0.15464 -0.15647 2.95266
C -2.19094 2.29299 3.44572
H -1.87259 2.07214 2.42051
H -3.19592 2.725 3.37781
C -2.24529 0.97939 4.24506
H -2.78084 1.16943 5.19498
H -2.83855 0.22148 3.71891

^{3}PC$_{reb}$

Fe 0.00208 -0.21817 -0.05059
O -0.19938 -0.66287 2.18501
N 2.05243 -0.23705 -0.13496
N 0.1776 -0.31668 -2.31399
N 0.32664 1.755 0.26502
N -0.30525 -2.23257 -0.44436
C 1.10148 1.46803 -5.06433
H 1.91811 1.8361 -4.44817
C 1.23083 1.46263 -6.45417
H 2.14998 1.81533 -6.91256
C 0.17685 1.01131 -7.25118
H 0.27421 1.00796 -8.33277
C -1.00817 0.5755 -6.65306
H -1.83677 0.23704 -7.26814
C -1.13372 0.58212 -5.26349
H -2.06426 0.25662 -4.80637
C -0.07668 1.01802 -4.45292
C -0.18892 0.98361 -2.94606
H 0.45488 1.7485 -2.50619
H -1.21328 1.20213 -2.63094
C 1.5872 -0.70284 -2.52764
H 1.90763 -0.50423 -3.55669
H 1.66918 -1.7819 -2.37713
C 2.51363 0.01409 -1.55545
H 2.46698 1.09088 -1.73022
C 3.96514 -0.52411 -1.63511
H 4.08417 -1.13809 -2.53225
H 4.68568 0.29316 -1.70632
C 4.17518 -1.35845 -0.34541
H 4.61509 -2.33721 -0.54829
H 4.83836 -0.83942 0.35198
C 2.76675 -1.49723 0.24352
H 2.23592 -2.33113 -0.21487
H 2.73366 -1.62987 1.32614
C 2.52486 0.88879 0.72141
H 3.55589 1.17012 0.4888
H 2.51102 0.55171 1.76088
C 1.60541 2.06476 0.5722
C 2.00947 3.37872 0.7815
H 3.04535 3.59331 1.01901
C 1.06348 4.3984 0.68288
H 1.35444 5.43114 0.8427
C -0.25686 4.07348 0.37136
H -1.02175 4.8359 0.28303
C -0.58573 2.7397 0.16748
H -1.59483 2.43645 -0.07945
C -0.87582 -3.86731 -2.09429
H -1.11495 -4.09056 -3.12835
C -0.80945 -4.87611 -1.13566
H -0.99947 -5.90807 -1.41159
C -0.48877 -4.53824 0.17896
H -0.4233 -5.28916 0.95782
C -0.24144 -3.20557 0.48346
H 0.0116 -2.88206 1.48528
C -0.62706 -2.55049 -1.71583
C -0.73348 -1.40647 -2.69654
H -0.55136 -1.7661 -3.71655
H -1.75544 -1.01323 -2.66979
C -3.09992 -0.01949 -0.04086
C -4.55116 0.04352 -0.06296
H -4.9656 -0.88194 0.34709
H -4.89288 0.89112 0.53787
H -4.89631 0.16808 -1.0935
N -1.94397 -0.0663 -0.02907
C 0.22984 1.0772 3.86976
H 0.56744 1.76347 3.0868
H -0.85236 1.23165 3.98924
C 0.94417 1.37333 5.19762
H 2.03065 1.30549 5.04424
H 0.73368 2.40312 5.50723
C 0.46817 -0.36852 3.43898
H -1.15901 -0.64503 2.34253
H 1.5291 -0.52174 3.21866
C 0.04082 -1.36335 4.51524
H 0.25215 -2.38383 4.17594
H -1.04747 -1.28371 4.65589
C 0.52207 0.38272 6.29103
H -0.54391 0.5268 6.51725
H 1.07347 0.58447 7.21678
C 0.75248 -1.06754 5.8448
H 1.8304 -1.24361 5.72219
H 0.40417 -1.76748 6.61249

^{5}PC$_{reb}$

Fe 0.13094 -0.12034 0.14245
O -0.1469 -0.43023 2.26676
N 2.35361 -0.0657 -0.08396
N 0.2462 -0.29449 -2.0984
N 0.58636 1.98911 0.34303
N -0.24572 -2.21905 -0.21342
C 1.03683 1.38919 -4.95233

H 1.89954 1.74949 -4.39763
C 1.08265 1.34715 -6.34674
H 1.98232 1.66219 -6.86696
C -0.02883 0.90826 -7.0691
H 0.00407 0.87671 -8.1541
C -1.18789 0.52201 -6.39165
H -2.06095 0.19378 -6.94791
C -1.22976 0.56469 -4.99775
H -2.13989 0.27636 -4.4787
C -0.11469 0.9875 -4.26117
C -0.14438 0.99529 -2.75007
H 0.52587 1.76585 -2.36525
H -1.1484 1.22901 -2.38551
C 1.64573 -0.69148 -2.37957
H 1.87854 -0.57884 -3.44445
H 1.74159 -1.75504 -2.14728
C 2.65623 0.09449 -1.54837
H 2.58668 1.15674 -1.79154
C 4.09584 -0.45003 -1.7554
H 4.12481 -1.11246 -2.62541
H 4.79944 0.36301 -1.94672
C 4.44361 -1.2094 -0.4499
H 4.85812 -2.20194 -0.64052
H 5.17908 -0.65338 0.13859
C 3.10765 -1.29325 0.3017
H 2.52744 -2.1599 -0.02407
H 3.20237 -1.34617 1.38777
C 2.83812 1.11605 0.66257
H 3.84478 1.41791 0.35201
H 2.90208 0.84328 1.72108
C 1.89027 2.28305 0.52161
C 2.33031 3.59983 0.63201
H 3.38705 3.80519 0.76296
C 1.39677 4.63306 0.57565
H 1.71844 5.66568 0.66126
C 0.04761 4.322 0.40254
H -0.71016 5.09508 0.35172
C -0.31455 2.98628 0.28599
H -1.34651 2.68869 0.14264
C -0.80742 -3.85433 -1.86331
H -1.04915 -4.08289 -2.89554
C -0.72999 -4.8603 -0.90119
H -0.91386 -5.89382 -1.17576
C -0.40664 -4.52299 0.41372
H -0.3331 -5.27585 1.18987
C -0.16998 -3.18776 0.71606
H 0.08391 -2.86049 1.71726
C -0.56429 -2.53681 -1.48429
C -0.68049 -1.38674 -2.45718
H -0.51998 -1.74015 -3.48214
H -1.69928 -0.98797 -2.40751
C -3.18485 0.25872 0.10052
C -4.63612 0.27164 0.03162
H -4.98736 1.30009 -0.0914
H -4.96741 -0.32832 -0.82082
H -5.05281 -0.14583 0.95258
N -2.02793 0.25262 0.14869
C -0.60773 1.67328 4.43498
H -0.51962 2.70924 4.77973
H -1.35956 1.68622 3.63147
C -1.08423 0.76311 5.57603
H -0.3877 0.85621 6.421
H -2.0669 1.0882 5.93636
C 0.74348 1.20363 3.87551
H 1.0529 1.83368 3.03704
H 1.51362 1.29844 4.65183
C 0.70413 -0.26363 3.44294
H 1.68494 -0.59394 3.09407
H -1.06699 -0.22534 2.50208
C -1.14082 -0.70676 5.13528
H -1.93286 -0.83444 4.38143
H -1.42537 -1.35006 5.97489
C 0.20862 -1.17502 4.56679
H 0.96365 -1.15947 5.36294
H 0.1458 -2.20656 4.20245

^{2}FP

Fe 0.11855 0.00045 -0.07215
O 0.16744 -0.09477 1.72771
N 2.13457 0.04441 -0.1261
N 0.21568 -0.19951 -2.16466
N 0.32806 1.97711 -0.0103
N -0.0751 -1.97836 -0.23454
C 0.90058 1.2868 -5.16754
H 1.75454 1.72563 -4.65803
C 0.92385 1.14552 -6.55565
H 1.79982 1.4594 -7.11507
C -0.18082 0.61074 -7.22139
H -0.16524 0.50172 -8.30161
C -1.3124 0.23075 -6.49579
H -2.18139 -0.1689 -7.00961
C -1.33303 0.37289 -5.10841
H -2.22411 0.09249 -4.5538
C -0.22117 0.88752 -4.42746
C -0.23068 1.02443 -2.92419
H 0.42403 1.84046 -2.6191
H -1.23623 1.25167 -2.56611
C 1.64139 -0.55074 -2.44608
H 1.87105 -0.39177 -3.50241
H 1.76897 -1.61657 -2.2488
C 2.58026 0.24358 -1.55881
H 2.50768 1.30797 -1.78846
C 4.04022 -0.27051 -1.63446
H 4.14118 -0.97381 -2.46591
H 4.73695 0.54938 -1.81691
C 4.30876 -0.96395 -0.27518
H 4.81713 -1.9234 -0.3905
H 4.93265 -0.33725 0.36771
C 2.91412 -1.15018 0.33044
H 2.43723 -2.04062 -0.07689
H 2.88214 -1.20996 1.41823
C 2.50852 1.23856 0.68684
H 3.54542 1.53433 0.50748
H 2.3976 0.96193 1.73793
C 1.56586 2.36078 0.37517
C 1.90253 3.70355 0.50265
H 2.90892 3.97875 0.79653
C 0.9317 4.67055 0.24984
H 1.17182 5.72411 0.34355
C -0.3489 4.26572 -0.12802
H -1.13355 4.9842 -0.33246
C -0.6136 2.90924 -0.24955
H -1.58861 2.54866 -0.54596
C -0.66417 -3.78754 -1.67756
H -0.94517 -4.12833 -2.6674
C -0.51661 -4.68136 -0.61792
H -0.68189 -5.74227 -0.77197
C -0.15381 -4.19545 0.63866
H -0.03251 -4.85759 1.48773
C 0.0591 -2.8321 0.79542
H 0.33491 -2.38 1.73954
C -0.4466 -2.4336 -1.44919

C -0.64128 -1.37153 -2.49389
H -0.42068 -1.74977 -3.49599
H -1.68429 -1.04403 -2.48704
C -2.99682 0.11402 -0.01697
C -4.44604 0.10422 0.06493
H -4.78692 -0.88641 0.37907
H -4.77638 0.85095 0.79265
H -4.86852 0.34041 -0.91594
N -1.84424 0.12752 -0.0908
H -0.55992 0.43542 2.09803

^{6}**FP**

Fe 0.1566 -0.2493 0.44303
O 0.23534 -0.63641 2.23598
N 2.30771 -0.19847 0.13713
N 0.23606 -0.27278 -1.85061
N 0.58247 1.86059 0.58537
N -0.26522 -2.26862 -0.08723
C 1.01733 1.47041 -4.66808
H 1.91343 1.76428 -4.12759
C 1.01972 1.48316 -6.06362
H 1.91932 1.77154 -6.59891
C -0.13491 1.13444 -6.76708
H -0.13553 1.14582 -7.8529
C -1.29363 0.78459 -6.06973
H -2.19958 0.52835 -6.61075
C -1.29271 0.77199 -4.67482
H -2.2031 0.51517 -4.14013
C -0.13403 1.10162 -3.95827
C -0.1223 1.05882 -2.44842
H 0.58964 1.78642 -2.05787
H -1.10585 1.31686 -2.0499
C 1.62431 -0.69119 -2.17551
H 1.8406 -0.52092 -3.23487
H 1.69683 -1.76824 -2.00773
C 2.65261 0.02416 -1.31021
H 2.62445 1.09801 -1.5024
C 4.07403 -0.56473 -1.50582
H 4.09372 -1.20021 -2.39561
H 4.80868 0.2282 -1.65785
C 4.36936 -1.3777 -0.21935
H 4.76941 -2.37054 -0.43527
H 5.09767 -0.85869 0.40987
C 3.01396 -1.46934 0.49274
H 2.42399 -2.3025 0.10647
H 3.06593 -1.56646 1.57718
C 2.78771 0.93065 0.97398
H 3.82132 1.19504 0.73159
H 2.76161 0.60209 2.01741
C 1.8872 2.1266 0.81164
C 2.3509 3.43262 0.92858
H 3.40666 3.61597 1.09299
C 1.44152 4.48463 0.82985
H 1.78243 5.51079 0.91612
C 0.09265 4.20145 0.61415
H -0.64622 4.98961 0.53152
C -0.2978 2.87463 0.49455
H -1.33005 2.60154 0.31706
C -0.93655 -3.80906 -1.7809
H -1.21064 -3.9884 -2.8144
C -0.88306 -4.85092 -0.85583
H -1.11869 -5.8644 -1.16293
C -0.51813 -4.57834 0.46381
H -0.46403 -5.36268 1.20944
C -0.2143 -3.26978 0.81277
H 0.07637 -2.98414 1.81679
C -0.62689 -2.51954 -1.36173
C -0.71382 -1.32494 -2.2766
H -0.54237 -1.62353 -3.31571
H -1.72455 -0.91022 -2.22057
C -3.10211 0.24374 0.27562
C -4.55027 0.31614 0.31304
H -4.91305 0.83582 -0.57843
H -4.96432 -0.69594 0.33917
H -4.86481 0.86165 1.20742
N -1.94695 0.19302 0.23855
H 0.12067 0.10133 2.85892

CYH + **2'**

^{3}**RC**

Fe -0.00167 0.01522 0.02068
O 0.00087 0.01862 1.64914
N 0.33858 0.01045 -2.08516
N 0.7815 1.88701 -0.04074
N 1.71596 -1.01116 -0.06457
N -1.70086 1.03387 -0.07189
C 3.56456 3.83896 -0.38603
H 3.80441 3.19887 -1.23125
C 4.09404 5.12856 -0.32677
H 4.72787 5.4887 -1.13127
C 3.81574 5.94761 0.76929
H 4.22819 6.95086 0.81764
C 3.01835 5.46797 1.81113
H 2.81361 6.09378 2.67437
C 2.49035 4.1785 1.7502
H 1.88742 3.80616 2.57356
C 2.74439 3.35624 0.64363
C 2.16443 1.96575 0.56738
H 2.80743 1.32137 -0.03052
H 2.07137 1.52509 1.56019
C 0.76545 2.33468 -1.46978
H 1.40092 3.21515 -1.58813
H -0.25325 2.63842 -1.71606
C 1.20293 1.21184 -2.38861
H 2.24008 0.94126 -2.18403
C 0.97563 1.55029 -3.8847
H 0.72438 2.60923 -3.99229
H 1.87979 1.37039 -4.4687
C -0.19535 0.6453 -4.34127
H -0.92814 1.18415 -4.94529
H 0.16914 -0.19528 -4.93789
C -0.81501 0.15225 -3.02984
H -1.49583 0.90051 -2.62421
H -1.35117 -0.79507 -3.1008
C 1.0995 -1.23238 -2.3843
H 1.62124 -1.16392 -3.34309
H 0.38622 -2.05837 -2.45793
C 2.06543 -1.50641 -1.26986
C 3.22266 -2.26072 -1.43176
H 3.48724 -2.6305 -2.41583
C 4.01532 -2.53024 -0.31811
H 4.92168 -3.11696 -0.42168
C 3.62601 -2.03974 0.92893
H 4.20827 -2.23397 1.82175
C 2.4665 -1.28297 1.01883
H 2.10575 -0.87784 1.95546
C -2.68345 3.14329 0.46882
H -2.55408 4.16249 0.81397
C -3.93962 2.64845 0.12175
H -4.81647 3.28184 0.1987
C -4.05283 1.33475 -0.33353
H -5.00884 0.91339 -0.61971

C -2.90869 0.55374 -0.41871
H -2.94379 -0.4672 -0.77213
C -1.57967 2.3059 0.36625
C -0.18656 2.70894 0.74515
H -0.01443 3.77473 0.57691
H -0.02322 2.49224 1.80375
C -1.38173 -2.78313 -0.06222
N -0.90752 -1.73236 -0.11332
C -1.98338 -4.10201 -0.00127
H -1.58501 -4.72273 -0.8089
H -1.74743 -4.56318 0.96201
H -3.06827 -4.01572 -0.10886
C -3.04442 0.42226 3.40992
H -3.2022 1.39189 3.899
H -2.44162 0.61261 2.51318
C -4.39025 -0.1898 2.9966
H -5.02776 -0.29565 3.88647
H -4.91848 0.4819 2.30909
C -2.25864 -0.52128 4.33153
H -1.28324 -0.08317 4.57673
H -2.80331 -0.63775 5.28017
C -2.06977 -1.90116 3.68472
H -1.54013 -2.57435 4.37089
H -1.43437 -1.78622 2.79608
C -4.20087 -1.57001 2.35217
H -3.64835 -1.45038 1.40922
H -5.17258 -2.00783 2.09208
C -3.41646 -2.51378 3.27465
H -4.01374 -2.70955 4.17708
H -3.26414 -3.48515 2.78679

^{5}RC

Fe 0.51754 0.02798 0.59442
O 0.97573 0.05245 2.14924
N 0.28244 0.06159 -1.54841
N 1.21244 2.10802 0.24163
N 2.17316 -1.02925 0.00898
N -1.2213 1.16241 0.97537
C 3.77697 4.01396 -0.94031
H 3.80211 3.32382 -1.7797
C 4.27411 5.30847 -1.09778
H 4.66866 5.62141 -2.05969
C 4.27095 6.19367 -0.01803
H 4.65899 7.20063 -0.13843
C 3.77991 5.77627 1.22144
H 3.78977 6.45497 2.06891
C 3.28366 4.48197 1.3767
H 2.91935 4.15954 2.3481
C 3.26529 3.59131 0.29438
C 2.69854 2.20082 0.4509
H 3.16477 1.52006 -0.26315
H 2.88601 1.80883 1.45249
C 0.78756 2.44874 -1.14123
H 1.32765 3.33028 -1.49939
H -0.27142 2.7131 -1.11315
C 0.99922 1.28027 -2.09321
H 2.06101 1.0381 -2.16035
C 0.37347 1.55242 -3.48533
H 0.12383 2.61282 -3.57728
H 1.07798 1.31599 -4.28459
C -0.89425 0.6634 -3.55657
H -1.76832 1.2121 -3.9128
H -0.74086 -0.1838 -4.2301
C -1.09973 0.18158 -2.116
H -1.63805 0.92805 -1.53283
H -1.62005 -0.77179 -2.01841
C 0.9385 -1.17819 -2.05719
H 1.15627 -1.10425 -3.12574
H 0.238 -2.00652 -1.91841
C 2.18715 -1.45611 -1.27367
C 3.2801 -2.14482 -1.78499
H 3.27793 -2.4654 -2.82034
C 4.36291 -2.40989 -0.94679
H 5.22625 -2.94512 -1.32713
C 4.32724 -1.97892 0.38066
H 5.14932 -2.16896 1.06009
C 3.21161 -1.28619 0.82717
H 3.11579 -0.91824 1.8415
C -2.00908 3.30085 1.67901
H -1.78974 4.33553 1.91682
C -3.30881 2.80307 1.75086
H -4.12497 3.44929 2.05514
C -3.54728 1.46935 1.41843
H -4.54266 1.04341 1.45462
C -2.47465 0.6777 1.03225
H -2.60252 -0.36094 0.75829
C -0.97797 2.44993 1.29426
C 0.46129 2.89758 1.24267
H 0.52287 3.96906 1.02923
H 0.92109 2.71977 2.21939
C -1.11109 -2.89714 0.84551
N -0.66308 -1.85307 0.62807
C -1.68509 -4.20134 1.12081
H -1.139 -4.96782 0.56362
H -1.61365 -4.40953 2.19215
H -2.73606 -4.20967 0.81803
C -1.3234 0.59893 4.69638
H -1.20049 1.57699 5.17893
H -1.05792 0.73082 3.64038
C -2.78368 0.13615 4.79835
H -3.07916 0.10219 5.85722
H -3.44598 0.86113 4.30951
C -0.36185 -0.42649 5.31353
H 0.67577 -0.09641 5.18042
H -0.5417 -0.48647 6.39716
C -0.55696 -1.81728 4.6926
H 0.11264 -2.54456 5.16937
H -0.27493 -1.77129 3.63174
C -2.97374 -1.25748 4.18309
H -2.77718 -1.20358 3.10283
H -4.01436 -1.58615 4.29651
C -2.01607 -2.27943 4.81122
H -2.27027 -2.40506 5.87388
H -2.14735 -3.2632 4.34161

^{3}TS

Fe -0.06022 0.19813 0.00724
O -0.05889 0.31368 1.75508
N 0.32963 0.03892 -2.05531
N 0.90233 1.99541 -0.11777
N 1.57948 -0.95406 0.0575
N -1.66777 1.35855 -0.14857
C 3.83668 3.70348 -0.47045
H 4.0395 3.00856 -1.28119
C 4.47025 4.94673 -0.455
H 5.14773 5.21669 -1.25938
C 4.23954 5.83475 0.59748
H 4.73343 6.80167 0.61229
C 3.38355 5.46959 1.63928
H 3.21376 6.14863 2.46937
C 2.75124 4.2264 1.62119
H 2.10103 3.94292 2.4441

C 2.95859 3.33549 0.55886
C 2.26484 1.99533 0.52989
H 2.87065 1.26955 -0.01149
H 2.10541 1.61568 1.53972
C 0.96212 2.35127 -1.56895
H 1.67872 3.16051 -1.72964
H -0.01861 2.73013 -1.86315
C 1.30801 1.13649 -2.4072
H 2.31062 0.78305 -2.16121
C 1.14271 1.40279 -3.92551
H 0.98223 2.4707 -4.09894
H 2.04082 1.11654 -4.4756
C -0.09001 0.57298 -4.36136
H -0.75787 1.13223 -5.01985
H 0.21546 -0.33172 -4.89415
C -0.78176 0.21774 -3.04151
H -1.40552 1.04479 -2.7014
H -1.39522 -0.68365 -3.07325
C 0.98772 -1.28157 -2.25253
H 1.52234 -1.32509 -3.20576
H 0.20738 -2.04755 -2.27793
C 1.91507 -1.55266 -1.10376
C 3.01587 -2.39823 -1.19247
H 3.27078 -2.85059 -2.14412
C 3.76639 -2.65035 -0.04558
H 4.62913 -3.30642 -0.09032
C 3.3909 -2.05197 1.15767
H 3.94174 -2.22884 2.07391
C 2.28804 -1.2086 1.17167
H 1.93438 -0.7169 2.06888
C -2.4323 3.60597 0.1497
H -2.20186 4.63497 0.40055
C -3.72539 3.21745 -0.19602
H -4.52847 3.94609 -0.21687
C -3.97009 1.88242 -0.51932
H -4.95925 1.53759 -0.79564
C -2.91346 0.98214 -0.49106
H -3.05045 -0.05899 -0.74901
C -1.42447 2.64818 0.1753
C -0.01132 2.94303 0.5819
H 0.26079 3.97958 0.36714
H 0.09289 2.7667 1.65505
C -1.68525 -2.45741 0.02177
N -1.12711 -1.44843 -0.03022
C -2.39829 -3.71976 0.09193
H -2.01801 -4.30694 0.9328
H -3.46576 -3.52916 0.23459
H -2.24943 -4.27774 -0.83698
C -2.70964 1.4866 3.28044
H -1.9515 2.17804 3.66486
H -3.01408 1.84864 2.29208
C -3.95677 1.48505 4.19468
H -3.65552 1.22297 5.21789
H -4.38144 2.49549 4.233
C -2.15501 0.08283 3.1658
H -1.12566 0.15382 2.34023
H -1.63226 -0.24356 4.07265
C -3.15417 -0.93815 2.66749
H -2.71166 -1.93985 2.64073
H -3.47281 -0.68588 1.64823
C -4.9979 0.47687 3.69194
H -5.36098 0.7955 2.70413
H -5.86673 0.46642 4.36128
C -4.40333 -0.93282 3.58094
H -4.11927 -1.29051 4.57977
H -5.14454 -1.63798 3.18577

[5]TS

Fe 0.07939 -0.29575 0.41335
N 0.26981 -0.13274 -1.81522
N 0.68216 1.80068 0.27729
N 1.93852 -1.23285 0.00913
N -1.81203 0.74822 0.49516
C 3.2949 3.91043 -0.29253
H 3.49815 3.28481 -1.15781
C 3.742 5.23233 -0.27632
H 4.2777 5.63135 -1.13235
C 3.50731 6.03466 0.84214
H 3.85625 7.06285 0.85756
C 2.83436 5.50711 1.94686
H 2.66294 6.12147 2.82563
C 2.3883 4.18555 1.9278
H 1.87991 3.77715 2.79688
C 2.60255 3.3767 0.80319
C 2.09703 1.95497 0.76991
H 2.72662 1.34542 0.12055
H 2.11508 1.5065 1.76474
C 0.50775 2.22569 -1.13776
H 1.03676 3.16628 -1.31781
H -0.55408 2.42229 -1.29781
C 0.98017 1.15894 -2.11757
H 2.05289 0.99219 -2.00129
C 0.60561 1.5276 -3.57786
H 0.31494 2.5804 -3.63481
H 1.45769 1.3922 -4.24667
C -0.5705 0.58987 -3.95264
H -1.40257 1.12707 -4.41295
H -0.24483 -0.17897 -4.65869
C -0.98701 -0.04283 -2.61797
H -1.68307 0.60475 -2.08307
H -1.44271 -1.03052 -2.70112
C 1.09146 -1.28572 -2.25843
H 1.50627 -1.13335 -3.26004
H 0.43802 -2.16261 -2.30599
C 2.19653 -1.55832 -1.27343
C 3.39994 -2.15443 -1.63759
H 3.59007 -2.39337 -2.6779
C 4.34164 -2.43309 -0.64827
H 5.28699 -2.89611 -0.91081
C 4.0572 -2.10772 0.67934
H 4.76399 -2.30984 1.47542
C 2.84153 -1.50503 0.9705
H 2.55394 -1.22055 1.97518
C -2.79715 2.8159 1.17635
H -2.66352 3.84099 1.50326
C -4.06894 2.26855 1.01707
H -4.95048 2.86659 1.22167
C -4.19312 0.94727 0.58569
H -5.16308 0.48554 0.44426
C -3.03756 0.22125 0.32786
H -3.07694 -0.80299 -0.02181
C -1.68358 2.02138 0.91911
C -0.27707 2.51138 1.15528
H -0.21591 3.59436 1.0105
H -0.00117 2.2908 2.19068
C -1.3614 -3.2554 0.11049
N -0.94221 -2.17769 0.08968
C -1.89698 -4.60329 0.14543
H -1.07307 -5.32273 0.15723
H -2.50215 -4.72846 1.04805
H -2.51873 -4.77522 -0.73773
O 0.31298 -0.33729 2.11748

C -2.43515 0.48083 4.2768
H -2.94899 1.29445 4.80153
H -2.15089 0.87053 3.29249
C -3.37514 -0.71803 4.09063
H -3.73802 -1.05197 5.07314
H -4.25638 -0.41603 3.512
C -1.16239 0.08807 5.0397
H -0.46689 0.9331 5.10357
H -1.42672 -0.17637 6.07743
C -0.47493 -1.11715 4.42343
H 0.45729 -1.39574 4.92672
H -0.07568 -0.74353 3.33287
C -2.66083 -1.8814 3.39001
H -2.38939 -1.57316 2.37386
H -3.33211 -2.7427 3.29116
C -1.38523 -2.29823 4.13599
H -1.66062 -2.74673 5.10519
H -0.84227 -3.07211 3.58052

^{3}IM

Fe -0.03257 0.18008 0.06956
O 0.01177 0.29932 1.86172
N 0.2802 0.03747 -1.98804
N 0.89837 1.98886 -0.05912
N 1.62059 -0.94593 0.06383
N -1.66115 1.31093 -0.02635
C 3.7933 3.75298 -0.46903
H 3.98817 3.07133 -1.29283
C 4.40508 5.00716 -0.45422
H 5.05667 5.29893 -1.27227
C 4.18622 5.87796 0.61498
H 4.66304 6.85342 0.62931
C 3.36454 5.48449 1.67399
H 3.20466 6.15003 2.51689
C 2.75414 4.23039 1.65652
H 2.13117 3.92513 2.49256
C 2.94886 3.35661 0.57767
C 2.27814 2.0045 0.54869
H 2.88089 1.29878 -0.02184
H 2.15486 1.60768 1.55675
C 0.90972 2.35527 -1.50866
H 1.61582 3.16974 -1.68826
H -0.08201 2.72978 -1.77014
C 1.23758 1.14746 -2.36555
H 2.2504 0.80052 -2.15498
C 1.01893 1.41938 -3.87572
H 0.84072 2.48629 -4.03757
H 1.90126 1.14601 -4.45703
C -0.21855 0.57849 -4.27579
H -0.9125 1.1335 -4.91032
H 0.07847 -0.32115 -4.82158
C -0.86521 0.21297 -2.93647
H -1.48162 1.0355 -2.57365
H -1.47393 -0.69196 -2.95035
C 0.9419 -1.2777 -2.21848
H 1.43804 -1.3097 -3.19252
H 0.16734 -2.05031 -2.21674
C 1.91652 -1.54313 -1.10872
C 3.01974 -2.37969 -1.23792
H 3.24244 -2.83161 -2.19775
C 3.81439 -2.62331 -0.119
H 4.6801 -3.27253 -0.19484
C 3.47924 -2.02646 1.09687
H 4.06533 -2.19816 1.99197
C 2.37066 -1.19215 1.15219
H 2.04287 -0.7037 2.06066
C -2.45952 3.53222 0.35821
H -2.24231 4.55865 0.63008
C -3.75524 3.12543 0.04515
H -4.5735 3.83666 0.07287
C -3.98373 1.79538 -0.31009
H -4.97417 1.43816 -0.56504
C -2.9097 0.91664 -0.33867
H -3.03242 -0.12015 -0.62015
C -1.43062 2.59715 0.32051
C -0.00911 2.91358 0.67905
H 0.23549 3.95709 0.46478
H 0.13339 2.72773 1.74588
C -1.63769 -2.48246 0.20534
N -1.0845 -1.47361 0.10975
C -2.33848 -3.74844 0.32171
H -2.05418 -4.4009 -0.50885
H -2.07034 -4.22773 1.2675
H -3.41787 -3.5746 0.29457
C -2.79188 1.27426 3.52816
H -2.0075 1.79266 4.08957
H -2.8085 1.71074 2.52003
C -4.18275 1.53481 4.1646
H -4.15529 1.23901 5.2217
H -4.40351 2.60887 4.13451
C -2.52742 -0.19841 3.44777
H -0.83681 0.0007 2.26052
H -1.9132 -0.65821 4.22042
C -3.57526 -1.03501 2.78206
H -3.33114 -2.10137 2.83648
H -3.65313 -0.76849 1.7174
C -5.27414 0.7371 3.43865
H -5.35791 1.09863 2.40365
H -6.24659 0.9133 3.91453
C -4.96195 -0.76529 3.42523
H -4.95956 -1.14796 4.45441
H -5.73461 -1.3174 2.87634

^{5}IM

Fe 0.18992 -0.27579 0.48873
N 0.2934 -0.1285 -1.77474
N 0.76811 1.83589 0.27699
N 2.05639 -1.19662 -0.02178
N -1.70737 0.76369 0.59853
C 3.3322 3.96664 -0.40988
H 3.50675 3.33627 -1.27804
C 3.76464 5.29349 -0.4221
H 4.26059 5.69138 -1.30229
C 3.56635 6.10243 0.69865
H 3.90388 7.13451 0.69189
C 2.94453 5.57657 1.83373
H 2.8015 6.19633 2.71381
C 2.51298 4.25002 1.84298
H 2.04397 3.8433 2.73469
C 2.69098 3.43416 0.71715
C 2.19845 2.00706 0.71369
H 2.80872 1.39967 0.0441
H 2.26053 1.566 1.71039
C 0.53674 2.24166 -1.13352
H 1.05087 3.18337 -1.34811
H -0.5319 2.42856 -1.25657
C 0.9813 1.16439 -2.11632
H 2.05899 1.00801 -2.03656
C 0.55302 1.51214 -3.56686
H 0.26184 2.56452 -3.62899
H 1.37962 1.36522 -4.26472
C -0.63674 0.57005 -3.88384

H -1.48897 1.10267 -4.31152
H -0.34087 -0.20351 -4.59768
C -0.99456 -0.05296 -2.52734
H -1.67242 0.59638 -1.97208
H -1.44809 -1.0436 -2.58364
C 1.1036 -1.27848 -2.24412
H 1.469 -1.13319 -3.26609
H 0.45666 -2.1615 -2.25237
C 2.25806 -1.53058 -1.31099
C 3.45054 -2.11403 -1.72831
H 3.59375 -2.36057 -2.77438
C 4.44309 -2.36907 -0.78317
H 5.38135 -2.82135 -1.08687
C 4.21863 -2.03357 0.5534
H 4.96654 -2.21679 1.31587
C 3.00898 -1.44577 0.89706
H 2.76404 -1.15474 1.91148
C -2.68448 2.84457 1.25208
H -2.54736 3.87526 1.55916
C -3.95853 2.29714 1.11045
H -4.83744 2.89999 1.31209
C -4.08848 0.97076 0.69714
H -5.06012 0.5096 0.56631
C -2.93591 0.23851 0.44452
H -2.98037 -0.78958 0.10742
C -1.57358 2.04595 0.99648
C -0.16337 2.54335 1.18788
H -0.11386 3.62673 1.04165
H 0.14971 2.32277 2.21268
C -1.2372 -3.24121 0.22571
N -0.82539 -2.16027 0.22385
C -1.76292 -4.5931 0.233
H -0.9344 -5.30552 0.28366
H -2.41099 -4.72575 1.10431
H -2.33929 -4.76754 -0.67998
O 0.5581 -0.29128 2.26262
C -2.64082 0.3892 4.26797
H -3.13168 1.23131 4.76843
H -2.30927 0.754 3.28918
C -3.62508 -0.76968 4.06656
H -4.01324 -1.09365 5.04275
H -4.48614 -0.43151 3.47806
C -1.41055 -0.0521 5.07945
H -0.65733 0.74414 5.11796
H -1.72306 -0.22011 6.12793
C -0.80939 -1.3247 4.56843
H 0.19295 -1.58501 4.90746
H -0.01912 -0.67364 2.97983
C -2.94683 -1.95438 3.36782
H -2.63827 -1.64401 2.36315
H -3.65131 -2.78408 3.23894
C -1.70983 -2.44194 4.14243
H -2.05254 -2.96649 5.05492
H -1.15187 -3.18943 3.56519

$^{3}PC_{reb}$

Fe 0.06613 0.38165 0.16789
O -0.14255 0.88533 2.36383
N 0.38425 0.06228 -1.98262
N 1.05339 2.26026 -0.20557
N 1.71178 -0.75932 0.21025
N -1.52845 1.56751 0.03472
C 3.97604 3.88136 -0.9025
H 4.14705 3.08081 -1.61764
C 4.60668 5.11348 -1.08198
H 5.25263 5.26911 -1.94087
C 4.41291 6.1387 -0.15399
H 4.90446 7.09733 -0.29053
C 3.59572 5.92262 0.95823
H 3.45357 6.71045 1.69195
C 2.9666 4.68999 1.13433
H 2.34548 4.52421 2.01034
C 3.13864 3.65972 0.19969
C 2.43511 2.33391 0.37231
H 3.01672 1.53756 -0.09348
H 2.33284 2.09158 1.43302
C 1.03979 2.41779 -1.6853
H 1.73699 3.20307 -1.99302
H 0.04065 2.75227 -1.97335
C 1.3579 1.11633 -2.41645
H 2.36417 0.78202 -2.15599
C 1.18012 1.27353 -3.95181
H 1.06196 2.33037 -4.2097
H 2.05687 0.90609 -4.48901
C -0.0907 0.46072 -4.30401
H -0.76208 1.00185 -4.9748
H 0.17403 -0.48138 -4.79298
C -0.74564 0.19375 -2.94181
H -1.35627 1.04493 -2.63195
H -1.37146 -0.69978 -2.90285
C 0.99317 -1.28403 -2.0476
H 1.48996 -1.47543 -3.0059
H 0.1864 -2.01732 -1.94489
C 1.96949 -1.46235 -0.91628
C 3.06642 -2.31451 -1.0094
H 3.2482 -2.85136 -1.93386
C 3.91359 -2.46025 0.08668
H 4.77185 -3.12156 0.03367
C 3.64569 -1.73213 1.24537
H 4.27987 -1.8018 2.12125
C 2.5407 -0.89236 1.26222
H 2.30801 -0.29645 2.13266
C -2.2913 3.83902 0.01742
H -2.05302 4.89392 0.09407
C -3.59805 3.409 -0.20725
H -4.40276 4.12987 -0.30485
C -3.85162 2.04173 -0.31423
H -4.85057 1.66285 -0.49546
C -2.79125 1.15354 -0.19133
H -2.93421 0.08383 -0.27423
C -1.27749 2.89488 0.13969
C 0.15482 3.26378 0.41575
H 0.37127 4.27789 0.06395
H 0.32627 3.23797 1.49549
C -1.5553 -2.34676 0.72611
N -1.02713 -1.34771 0.48145
C -2.21627 -3.59904 1.04891
H -1.662 -4.43288 0.60905
H -2.24879 -3.72008 2.1358
H -3.23648 -3.59179 0.65504
C -0.33192 1.21661 4.77639
H -0.05423 2.2677 4.63907
H -1.4283 1.15258 4.70288
C 0.1159 0.70199 6.15369
H 1.19845 0.85741 6.26085
H -0.36677 1.29238 6.94037
C 0.28194 0.37621 3.66006
H -1.1016 1.03931 2.41168
H 1.36662 0.52309 3.66849
C -0.04664 -1.10636 3.81299
H 0.42563 -1.67907 3.00812
H -1.13305 -1.23514 3.70848

C -0.20335 -0.78953 6.32371
H -1.29371 -0.92865 6.32719
H 0.16538 -1.14523 7.29284
C 0.4077 -1.62086 5.18771
H 1.50407 -1.56893 5.24716
H 0.13632 -2.67721 5.29315

$^5PC_{reb}$

Fe -0.11642 -0.3345 -0.00539
N 0.42613 -0.06753 -2.14357
N 0.66835 1.79997 0.09577
N 1.8068 -1.32809 -0.14291
N -1.90625 0.87014 0.14727
C 3.42826 3.76525 -0.26932
H 3.65213 3.13204 -1.12404
C 3.9496 5.0587 -0.21399
H 4.56461 5.42813 -1.02916
C 3.68656 5.87077 0.89117
H 4.09316 6.87666 0.93727
C 2.90969 5.38028 1.94345
H 2.71452 6.00112 2.81279
C 2.38978 4.08695 1.88439
H 1.80106 3.70666 2.71491
C 2.63251 3.26892 0.77248
C 2.04062 1.88079 0.69169
H 2.68558 1.23053 0.09854
H 1.97174 1.43979 1.68949
C 0.62973 2.25385 -1.31772
H 1.17082 3.19924 -1.43583
H -0.41412 2.45611 -1.56966
C 1.18354 1.21894 -2.2925
H 2.234 1.02137 -2.06919
C 0.9828 1.67473 -3.76441
H 0.69874 2.73081 -3.79495
H 1.90705 1.57334 -4.33703
C -0.14077 0.76809 -4.32755
H -0.90492 1.33344 -4.86594
H 0.26934 0.0271 -5.01981
C -0.72006 0.07722 -3.08504
H -1.47389 0.706 -2.60433
H -1.17007 -0.89897 -3.27295
C 1.28112 -1.21905 -2.5107
H 1.82847 -1.04796 -3.44439
H 0.6258 -2.08152 -2.67154
C 2.24703 -1.54705 -1.39814
C 3.50672 -2.08824 -1.64249
H 3.84082 -2.23902 -2.66311
C 4.31881 -2.42624 -0.56103
H 5.30479 -2.84688 -0.72878
C 3.84974 -2.2136 0.73591
H 4.44933 -2.46379 1.60336
C 2.58646 -1.65985 0.90117
H 2.16834 -1.46977 1.88218
C -2.80908 2.99924 0.75341
H -2.63564 4.01723 1.08447
C -4.09884 2.53051 0.51033
H -4.95331 3.18397 0.65157
C -4.27494 1.21608 0.0762
H -5.26018 0.81435 -0.1295
C -3.15022 0.41965 -0.0961
H -3.22679 -0.60703 -0.43515
C -1.72969 2.13877 0.56844
C -0.31488 2.5677 0.88394
H -0.20775 3.64788 0.72946
H -0.12166 2.38285 1.94525
C -1.61201 -3.23813 -0.63736
N -1.18991 -2.19519 -0.3644
C -2.1425 -4.54502 -0.98667
H -1.52351 -4.99287 -1.76941
H -2.13777 -5.19311 -0.10589
H -3.16747 -4.4386 -1.35321
C -1.67258 0.2754 3.74136
H -2.09117 0.89721 2.94438
H -2.325 -0.60402 3.84179
C -1.63627 1.04453 5.07131
H -1.05634 1.96852 4.93646
H -2.65218 1.34795 5.34723
C 0.35947 -1.05487 4.45159
H 1.36822 -1.35706 4.14811
H -0.23493 -1.97213 4.57169
C -1.00108 0.20197 6.18548
H -1.64293 -0.66585 6.39284
H -0.944 0.78394 7.11264
C 0.39673 -0.28746 5.78306
H 1.06968 0.57557 5.68181
H 0.82105 -0.92809 6.56397
O -0.28962 -0.91701 2.09329
H -0.84957 -1.70793 2.18028
C -0.27341 -0.19911 3.35828
H 0.37237 0.66122 3.15892

^2FP

Fe 0.05292 -0.16656 0.27885
N 0.15199 -0.08876 -1.79811
N 0.73349 1.75423 0.24045
N 1.82449 -1.05325 0.0014
N -1.70295 0.73834 0.47294
C 3.31656 3.91944 -0.29796
H 3.49597 3.3397 -1.1997
C 3.76417 5.23926 -0.22631
H 4.27388 5.68294 -1.07614
C 3.5646 5.98139 0.93941
H 3.91411 7.00778 0.99798
C 2.9277 5.39507 2.03583
H 2.78507 5.96158 2.95107
C 2.48114 4.07576 1.96143
H 2.00184 3.62146 2.82406
C 2.65697 3.32823 0.78873
C 2.16237 1.90502 0.6981
H 2.77788 1.3389 0.00003
H 2.21059 1.41244 1.6697
C 0.52861 2.24414 -1.15768
H 1.10063 3.1601 -1.32469
H -0.52655 2.50056 -1.27194
C 0.90408 1.17324 -2.16345
H 1.97164 0.95637 -2.1002
C 0.46993 1.53824 -3.60636
H 0.12978 2.57721 -3.63833
H 1.30449 1.44845 -4.30375
C -0.68145 0.56273 -3.95482
H -1.51269 1.06173 -4.45706
H -0.33214 -0.23787 -4.61254
C -1.11193 0.00175 -2.59687
H -1.78078 0.69808 -2.09118
H -1.59585 -0.97501 -2.63222
C 0.9492 -1.27331 -2.22609
H 1.33416 -1.1498 -3.2422
H 0.28695 -2.14368 -2.22792
C 2.06605 -1.49685 -1.24931
C 3.2481 -2.15567 -1.56872
H 3.42282 -2.48402 -2.58684
C 4.18439 -2.38458 -0.56189

H 5.11389 -2.89653 -0.78682
C 3.90814 -1.95225 0.73557
H 4.60496 -2.11998 1.54823
C 2.71344 -1.28912 0.98238
H 2.42696 -0.9351 1.96425
C -2.71677 2.79217 1.16018
H -2.59618 3.81378 1.50188
C -3.98033 2.24144 0.95439
H -4.87078 2.83285 1.13817
C -4.08414 0.92556 0.5023
H -5.04671 0.46093 0.32527
C -2.92188 0.20407 0.26933
H -2.94955 -0.81513 -0.0909
C -1.59312 2.01025 0.91865
C -0.18977 2.48404 1.15563
H -0.1033 3.56485 1.01818
H 0.09931 2.23473 2.17905
C -1.17892 -3.02599 0.285
N -0.77182 -1.94606 0.24845
C -1.69832 -4.38119 0.31988
H -0.86695 -5.08914 0.38198
H -2.3458 -4.50252 1.19293
H -2.27449 -4.57475 -0.58948
O 0.26582 -0.20594 2.07022
H -0.47293 -0.69054 2.47775

[6]FP

Fe 0.06729 -0.27346 0.4869
N 0.24039 -0.13115 -1.75938
N 0.68541 1.82926 0.29153
N 1.93616 -1.20955 0.05193
N -1.80774 0.78111 0.61324
C 3.29536 3.92084 -0.32454
H 3.47765 3.29134 -1.1916
C 3.75023 5.24011 -0.32148
H 4.27048 5.63348 -1.18953
C 3.54346 6.04691 0.79929
H 3.89855 7.07307 0.80441
C 2.89085 5.52642 1.91941
H 2.7415 6.14417 2.7998
C 2.4367 4.20748 1.91359
H 1.94461 3.80452 2.79449
C 2.62272 3.39453 0.78702
C 2.10776 1.97587 0.76684
H 2.72558 1.35997 0.11253
H 2.13546 1.53261 1.76428
C 0.49803 2.23668 -1.12573
H 1.03211 3.17027 -1.32582
H -0.56418 2.43993 -1.27629
C 0.9516 1.15197 -2.09363
H 2.02514 0.98276 -1.99015
C 0.55757 1.49633 -3.55404
H 0.28224 2.55212 -3.62767
H 1.39624 1.33402 -4.23369
C -0.6378 0.56836 -3.89034
H -1.4746 1.11013 -4.33631
H -0.33793 -0.21164 -4.59539
C -1.03167 -0.04654 -2.54043
H -1.71668 0.60896 -2.00059
H -1.49186 -1.03318 -2.60532
C 1.05232 -1.29215 -2.2004
H 1.45067 -1.1512 -3.21019
H 0.39765 -2.16855 -2.22845
C 2.17428 -1.55113 -1.23007
C 3.37348 -2.14733 -1.60635
H 3.54747 -2.39976 -2.64626
C 4.333 -2.40761 -0.62888
H 5.27608 -2.86972 -0.90094
C 4.06973 -2.06513 0.69871
H 4.79109 -2.2529 1.48512
C 2.85597 -1.46459 1.00237
H 2.58409 -1.17046 2.00899
C -2.7736 2.90721 1.12309
H -2.63119 3.95097 1.37925
C -4.05014 2.37127 0.95857
H -4.92589 2.99827 1.08733
C -4.18649 1.02475 0.62
H -5.16044 0.57102 0.48034
C -3.03883 0.26144 0.45287
H -3.08717 -0.78561 0.18123
C -1.66784 2.08119 0.95185
C -0.25672 2.56512 1.1691
H -0.18812 3.64435 1.00262
H 0.03221 2.36594 2.20538
C -1.36345 -3.21593 0.10603
N -0.96437 -2.1319 0.16583
C -1.86714 -4.57344 0.02105
H -1.08905 -5.22363 -0.38951
H -2.14524 -4.92319 1.01938
H -2.74393 -4.59701 -0.63244
O 0.37492 -0.36503 2.29796
H -0.40098 -0.33778 2.88324
